# Hyperubiquitylation of DNA helicase RECQL4 by E3 ligase MITOL prevents mitochondrial entry and potentiates mitophagy

**DOI:** 10.1016/j.jbc.2023.105087

**Published:** 2023-07-24

**Authors:** Mansoor Hussain, Aftab Mohammed, Shabnam Saifi, Swati Priya, Sagar Sengupta

**Affiliations:** 1 Signal Transduction Laboratory, National Institute of Immunology, New Delhi, India; 2 National Institute of Biomedical Genomics, Kalyani, India

**Keywords:** Rothmund–Thomson syndrome, mitochondrial replication, RecQ helicases, E3 ligases, autophagy

## Abstract

Mutations in the DNA helicase RECQL4 lead to Rothmund–Thomson syndrome (RTS), a disorder characterized by mitochondrial dysfunctions, premature aging, and genomic instability. However, the mechanisms by which these mutations lead to pathology are unclear. Here we report that RECQL4 is ubiquitylated by a mitochondrial E3 ligase, MITOL, at two lysine residues (K1101, K1154) *via* K6 linkage. This ubiquitylation hampers the interaction of RECQL4 with mitochondrial importer Tom20, thereby restricting its own entry into mitochondria. We show the RECQL4 2K mutant (where both K1101 and K1154 are mutated) has increased entry into mitochondria and demonstrates enhanced mitochondrial DNA (mtDNA) replication. We observed that the three tested RTS patient mutants were unable to enter the mitochondria and showed decreased mtDNA replication. Furthermore, we found that RECQL4 in RTS patient mutants are hyperubiquitylated by MITOL and form insoluble aggregate-like structures on the outer mitochondrial surface. However, depletion of MITOL allows RECQL4 expressed in these RTS mutants to enter mitochondria and rescue mtDNA replication. Finally, we show increased accumulation of hyperubiquitylated RECQL4 outside the mitochondria leads to the cells being potentiated to increased mitophagy. Hence, we conclude regulating the turnover of RECQL4 by MITOL may have a therapeutic effect in patients with RTS.

RECQL4, RECQL5, BLM, RECQL1, and WRN are the five RecQ helicase family members in human cells (1). Mutations in RECQL4 cause three different disorders with distinct yet overlapping phenotypes, namely, RAPADILINO, Baller–Gerold, and Rothmund–Thomson syndrome (RTS) (2). Patients with these disorders exhibit significant growth retardation and accelerated premature aging. In addition, patients with RTS have abnormal skeletal growth and radial ray defects (2, 3, 4). RECQL4 has been implicated in multiple cellular functions including the maintenance of genomic integrity (5).

Unlike other RecQ helicases, RECQL4 is present in both nucleus and mitochondria (6, 7). RECQL4 is constitutively present in the mitochondria in asynchronously growing cells. Only in the S-phase or when cells are exposed to genotoxic stress, RECQL4 localizes to the nucleus and participates in repair pathways (7). In mitochondria RECQL4 promotes mitochondrial DNA replication by its interaction with mitochondrial polymerase PolγA. Interaction of RECQL4 with PolγA enhanced the latter’s catalytic activity and allowed PolγA to bind better with the mitochondrial DNA (mtDNA) (7, 8). Consequently, RTS patient cells lacking RECQL4 have mutations incorporated in their mtDNA that are associated with aging and/or cancer (8). Human cells lacking mitochondrial RECQL4 exhibit increased reactive oxygen species, mtDNA mutations, and altered metabolism phenotype that leads to cancer predisposition (9).

Mitochondria are vital organelles for energy production in eukaryotic cells, ATP generation through oxidative phosphorylation (10). A vast majority of the mitochondrial proteins are encoded in the nuclear genome, synthesized as precursors in cytosol, and transported into mitochondria through protein import machinery. Most precursor mitochondrial proteins containing the mitochondrial localization signal (MLS) are recognized by Tom20 and enter into the mitochondrial matrix using a complex machinery involving multiple proteins and membrane potential (11). Recent studies have implicated the role of ubiquitylation in mitochondrial protein import (12). Mitochondrial complexome studies have revealed the quality control pathway of protein import and have demonstrated preprotein ubiquitylation, deubiquitylation, and degradation are interregulatory steps in this process (13).

MITOL/MARCH5/RNF153 (hereafter called MITOL) is a mitochondrial E3 ligase present on the mitochondrial outer membrane. It is an important regulator of mitochondrial dynamics and quality control (14). MITOL regulates mitochondrial dynamics by controlling the levels of mitochondrial fission factors like DRP1 and Mid49 (15, 16, 17) and fusion factor, Mitofusin 1 (MFN1) (18). Hence MITOL can regulate cellular proliferation *versus* senescence by controlling the levels of MFN1 and DRP1 (19). Others and we have recently demonstrated that MITOL can regulate mitochondrial protein import by coordinating with the mitochondrial deubiquitinase, USP30 (20, 21). Specifically we have provided evidence that nonubiquitylated PolγA enters mitochondria and positively regulates mtDNA replication. A subset of PolγA mutants seen in patients with Progressive External Ophthalmoplegia (PEO) undergo hyperubiquitylation and cannot enter the mitochondrial matrix, leading to compromised mtDNA replication (20). In an effort to determine whether other components of mitochondrial replication machinery are also regulated by MITOL, we investigated whether mitochondrial helicase, RECQL4, is also targeted by this E3 ligase. Here we provide evidence that the entry of RECQL4 into mitochondria is also regulated by MITOL *via* ubiquitylation at specific residues. Hyperubiquitylation of RTS mutants by MITOL leads to formation of insoluble aggregate-like structures on the outer mitochondrial membrane, leading to compromised mitochondrial replication and causing the cells to undergo mitophagy.

## Results

### MITOL causes the turnover of mitochondrial RECQL4

**Figure 1:**
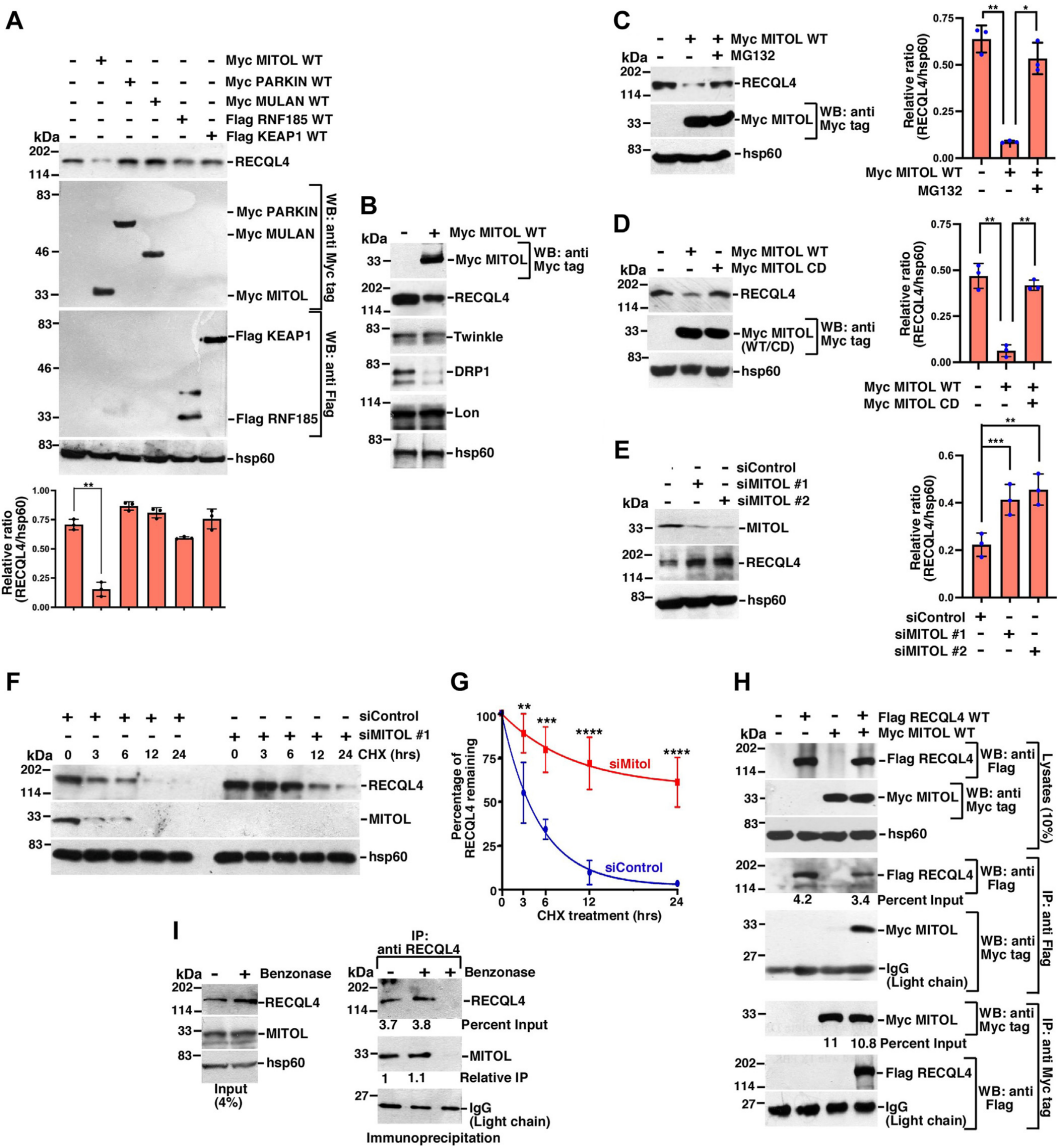
**MITOL negatively regulates the turnover of RECQL4.***A* and *B*, MITOL expression decreases the level of endogenous RECQL4. (*A*, *top*) Five Myc- or Flag-tagged mitochondrial E3 ligases are overexpressed in HEK293T cells. *B*, only Myc-MITOL was overexpressed. Western blot analysis was carried out using whole cell lysates with the indicated antibodies. The graph (*A*, *bottom*) represents quantification of the relative levels of RECQL4 to hsp60 from three replicates. *C*, RECQL4 undergoes proteasomal degradation. Myc-MITOL was expressed in HEK293T cells in absence or presence of MG132. (*Left*) Western blots were carried out using whole cell lysates with the indicated antibodies. (*Right*) Quantitation of relative levels of RECQL4 to hsp60 from three replicates. *D*, catalytic function of MITOL is required for proteasomal degradation of RECQL4. Either MITOL WT or MITOL CD was expressed in HEK293T cells. (*Left*) Western blots were carried out using whole cell lysates with the indicated antibodies. (*Right*) Quantitation of relative levels of RECQL4 to hsp60 from three replicates. *E*, ablation of MITOL stabilizes RECQL4 protein levels. HEK293T cells were transfected with siControl, siMITOL #1 and siMITOL #2. Western blots were carried out using whole cell lysates with the indicated antibodies. (*Right*) Quantitation of relative levels of RECQL4 to hsp60 from three replicates. *F* and *G*, half-life of RECQL4 increases upon MITOL ablation. *F*, HEK293T cells were transfected with siControl or siMITOL #1 for 24 h, after which the cells were treated with cycloheximide (CHX) for the indicated time periods. Western blots were carried out using whole cell lysates with the indicated antibodies. *G*, quantification of the percentage of RECQL4 remaining at each time point after CHX treatment. Data from three replicates. *H*, RECQL4 interacts with MITOL. (*Top*) HEK293T cells were transfected with either Myc-MITOL or Flag-RECQL4 or both. Western blots were carried out using whole cell lysates with the indicated antibodies. Immunoprecipitations using either (*Middle*) anti-Flag antibody or (*Bottom*) anti Myc antibody were carried out with the whole cell lysates. Western blots were carried out with the indicated antibodies. IgG (Light chain) indicated equal amount of antibody used for the immunoprecipitations. Three replicates were carried out and the same result was obtained. *I*, RECQL4–MITOL interaction is independent of the presence of nucleic acids. Lysates were made from asynchronously growing HEK293T cells. Lysates were either kept untreated or treated with benzonase. (*Left*) Western blots were carried out using the treated or untreated lysates with the indicated antibodies. (*Right*) Immunoprecipitation using anti-RECQL4 antibody was carried out and Western blots were carried out with the indicated antibodies.

In an effort to determine whether mitochondrial RECQL4 turnover occurs on the outer mitochondrial membrane, all five known mitochondrial surface E3 ligases (MITOL, PARKIN, MULAN, RNF185, KEAP1) were transiently overexpressed and endogenous levels of RECQL4 determined. While MITOL expression decreased the levels of RECQL4 (Fig. 1*A*) along with mitochondrial fission protein DRP1 (a known MITOL target (15, 17)), the levels of other key mitochondrial proteins like LONP protease and Twinkle protein levels remained unaltered (Fig. 1*B*). Treatment with proteasomal degradation inhibitor, MG132, reversed MITOL-dependent RECQL4 turnover (Fig. 1*C*).

In an effort to determine whether the catalytic activity of MITOL was involved in RECQL4 turnover, either wildtype MITOL (MITOL WT) or catalytically dead MITOL (MITOL CD) were overexpressed. Only MITOL WT was able to decrease RECQL4 protein levels (Fig. 1*D*). Hence ablation of MITOL by two different siRNAs (Fig. 1*E*) in a concentration gradient manner (Fig. S1*A*) led to an increase in the endogenous RECQL4 protein levels but no change was observed for RECQL4 transcripts (Fig. S1*B*). A similar increase in RECQL4 protein levels was also observed when MITOL was stably ablated in HeLa cells (22) (Fig. S1*C*) or Mitol^flox/flox^ MEFs were treated with 4-hydroxytamoxifen (22) (Fig. S1*D*). RECQL4 protein half-life increased upon MITOL ablation as determined by cycloheximide chase experiment (Fig. 1, *F* and *G*). Since RECQL4 is known to localize to both nucleus and mitochondria (6, 7), we wanted to determine whether MITOL specifically regulated the turnover of mitochondrial RECQL4. For this purpose, nuclear and mitochondrial fractions were isolated from cells overexpressing MITOL. It was found that MITOL only regulated the turnover of mitochondrial RECQL4 (Fig. S1*E*).

Next, we wanted to determine whether the turnover of RECQL4 by MITOL was due to their interaction in cells. Indeed, reciprocal immunoprecipitation experiments with the anti-Flag and anti-Myc tag antibodies revealed that RECQL4 interacts with MITOL (Fig. 1*H*). The interaction between RECQL4 and MITOL was not dependent on the presence of nucleic acids as similar interaction levels were seen in either the absence or presence of benzonase (Fig. 1*I*). This interaction occurred specifically on the mitochondrial surface as the mitoplast fraction did not show RECQL4–MITOL interaction (Fig. S1*F*). Hence MITOL interacted with RECQL4 WT but not RECQL4 lacking MLS (RECQL4 Δ84) (7) (Fig. S1*G*). Consequently, RECQL4, which cannot exit the nucleus due to lack of acetylation of five lysine residues (RECQL4 5K) (23), showed diminished interaction with MITOL (Fig. S1*H*). Colocalization of RECQL4 and MITOL was observed both for endogenous proteins (Fig. S2*A*) or when cells were transfected with Flag RECQL4 and Myc MITOL (Fig. S2*B*). *In vitro*–transcribed and translated RECQL4 interacted with GST-MITOL but not GST (Fig. S2*C*). Pull-down assays revealed that the C-terminal cytosol-facing loop region (253–278 aa) of MITOL interacted with RECQL4 (Fig. S2*D*), whereas the N terminus (1–459 aa) of RECQL4 interacted with MITOL (Fig. S2*E*).

### MITOL ubiquitylates RECQL4 *via* K6 linkage

**Figure 2:**
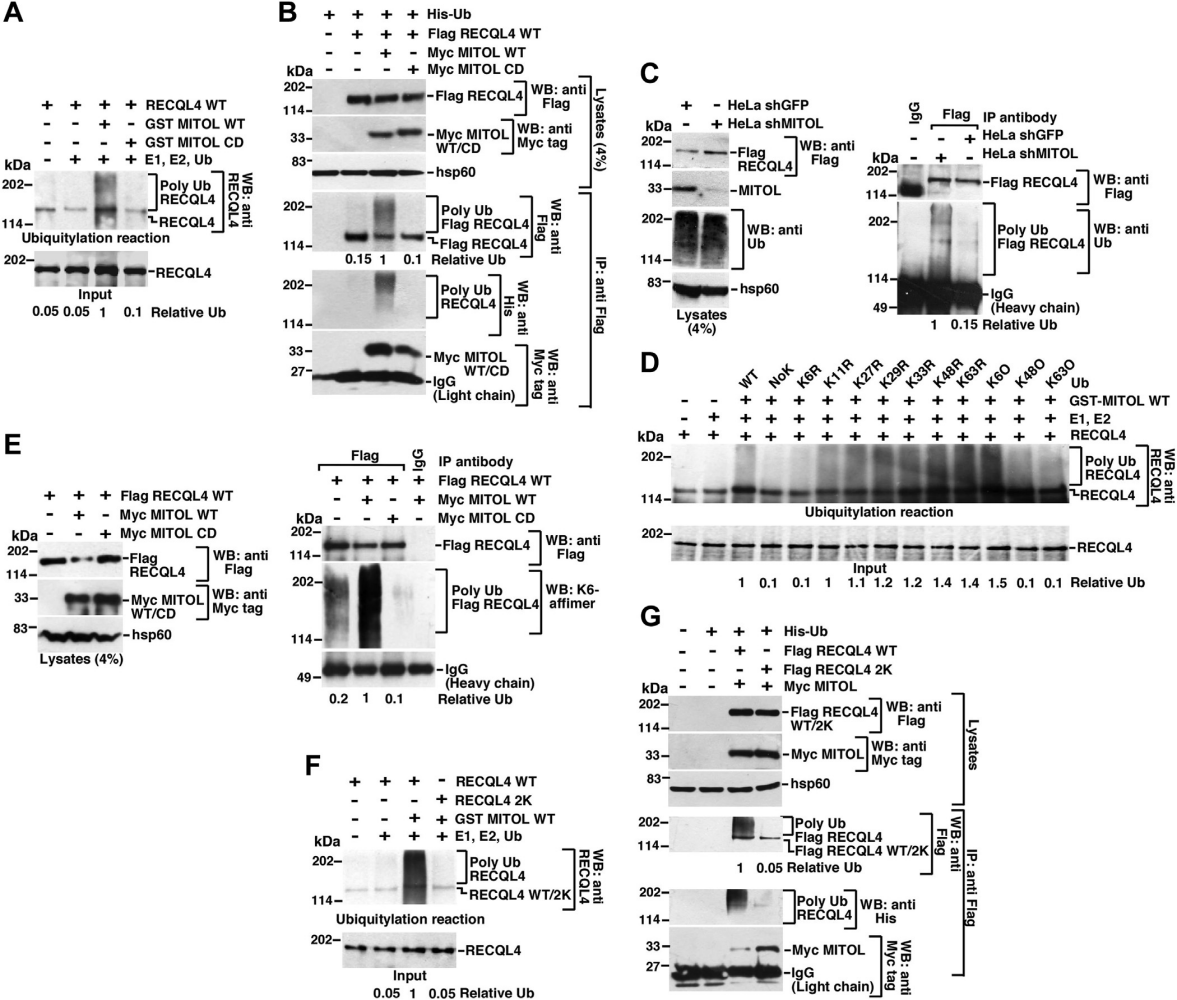
**RECQL4 is ubiquitylated by MITOL *via* K6-specific linkage at specific residues.***A*, MITOL ubiquitylates RECQL4 *in vitro*. *Invitro* ubiquitylation assays were performed, using S^35^-radiolabeled *in vitro–*transcribed and translated RECQL4 as the substrate and MITOL WT or CD as the E3 ligase, E1, E2 and wildtype ubiquitin. Ubiquitylated products were detected by Western blot with anti-RECQL4 antibody. Input indicates the amount of RECQL4 protein used in each ubiquitylation reaction. Five replicates were carried out and the same result was obtained. *B*, MITOL ubiquitylates RECQL4 in cells. HEK293T cells were transfected with Flag RECQL4, Myc-MITOL WT or CD, and His-Ubiquitin (His-Ub). (*Top*) Western blot analysis was carried out using whole cell lysates with the indicated antibodies. (*Bottom*) Immunoprecipitation using anti-Flag antibody was carried out with the whole cell lysates. Western blots were carried out with the indicated antibodies. Levels of Ig (Light chain) indicated equal amount of antibody used for the immunoprecipitations. Three replicates were carried out and the same result was obtained. *C*, MITOL ablation attenuates RECQL4 ubiquitylation in cells. HeLa shGFP and HeLa shMITOL were transfected with Flag-RECQL4. (*Left*) Western blot analysis were carried out using whole cell lysates with the indicated antibodies. (*Right*) Immunoprecipitation using anti-Flag antibody was carried out with the whole cell lysates. Western blots were carried out with the indicated antibodies. Levels of Ig (Light chain) indicated equal amount of antibody used for the immunoprecipitations. Three replicates were carried out and the same result was obtained. *D*, RECQL4 is ubiquitylated by MITOL *in vitro via* K6 linkage. *In vitro* ubiquitylation assay was performed and detected as in (*A*). In addition to wildtype Ubiquitin, Ubiquitin KR and KO mutants were also used. Three replicates were carried out and the same result was obtained. *E*, RECQL4 is ubiquitylated by MITOL in cells *via* K6-specific linkage. Ubiquitylation status of RECQL4 in cells was performed same as in (*B*), except His-Ub was not cotransfected. The extent of ubiquitylation on Flag-tagged immunoprecipitated RECQL4 was determined by Western blotting with K6 affimers. Three replicates were carried out and the same result was obtained. *F* and *G*, MITOL ubiquitylates RECQL4 at K1101, K1151. *F*, *in vitro* ubiquitylation assay was carried out and detected as in (*A*) using either RECQL4 WT or 2K as the substrate. *G*, ubiquitylation in cells on RECQL4 WT or RECQL4 2K was carried out and detected as in (*B*). Three replicates were carried out and the same result was obtained.

Since MITOL WT negatively regulates the turnover of mitochondrial RECQL4, we wanted to determine whether RECQL4 gets ubiquitylated by the E3 ligase. Indeed, *in vitro* ubiquitylation assays indicated that RECQL4 gets ubiquitylated by MITOL WT but not by MITOL CD (Fig. 2*A*). A progressive increase in the extent of ubiquitylation of RECQL4 by MITOL was observed with time (Fig. S3*A*). The effect of MITOL WT and not MITOL CD was also recapitulated when *in vivo* ubiquitylation assays were carried out (Fig. 2*B*). Consequently, ablation of MITOL in HeLa shMitol cells led to decreased ubiquitylation of RECQL4 (Fig. 2*C*).

Ubiquitylation of substrates occurs when ubiquitin moieties are linked to their substrates *via* one of the seven lysine residues (24). We wanted to determine the type of linkage *via* which RECQL4 gets ubiquitylated by MITOL. Hence *in vitro* ubiquitylation assays were carried out with wildtype (WT) ubiquitin, NoK ubiquitin, and its “R” and “O” variants (described in Experimental procedures). Only NoK and K6R ubiquitin variants led to the loss of ubiquitylation of RECQL4. Reciprocally the K6O ubiquitin variant showed ubiquitylation of RECQL4. Together the results indicated that MITOL ubiquitylated RECQL4 *via* K6 linkage (Fig. 2*D*). Usage of K6 linkage–specific affimers confirmed the K6 linkage on ubiquitylated RECQL4 WT *in vivo* (Fig. 2*E*). Unlike MITOL, ablation of HUWE1, an E3 ligase, which is known to ubiquitylate its substrate *via* K6 linkage (25), did not rescue RECQL4 protein levels (Fig. S3*B*). This indicates the specificity of RECQL4 ubiquitylation by MITOL.

Next, we wanted to determine the lysine residues on RECQL4 that are ubiquitylated by MITOL. Usage of ubiquitylation site prediction algorithms (UbPred and UbiPred) and PhosphoSitePlus server predicted four lysine residues (K695, K843, K1101, and K1154) on RECQL4 that could be potentially ubiquitylated by MITOL. Both *in vitro* (Fig. S3*C*) and *in vivo* (Fig. S3*D*) ubiquitylation assays revealed that the loss of K1101 and K1154 in RECQL4 led to decreased ubiquitylation of RECQL4. Complete loss of RECQL4 ubiquitylation was observed for RECQL4 2K mutant (where both K1101 and K1154 were mutated), both *in vitro* (Fig. 2*F*) and *in vivo* (Fig. 2*G*).

It has been reported that substrates ubiquitylated by MITOL are deubiquitylated by the mitochondrial deubiquitinase, USP30 (21). Therefore, we wanted to determine whether USP30 could deubiquitylate RECQL4 that has been ubiquitylated by MITOL. A MITOL-dependent RECQL4 *in vitro* ubiquitylation assay was carried out, and recombinant USP30 (Fig. S3*E*) was added either simultaneously or sequentially during the reaction. In both cases USP30 was not able to deubiquitylate MITOL-dependent ubiquitylation of RECQL4 (Fig. S3, *F* and *G*), thereby indicating that ubiquitylated RECQL4 is not a substrate of USP30.

### Ubiquitylation of RECQL4 compromises import and mtDNA replication

**Figure 3:**
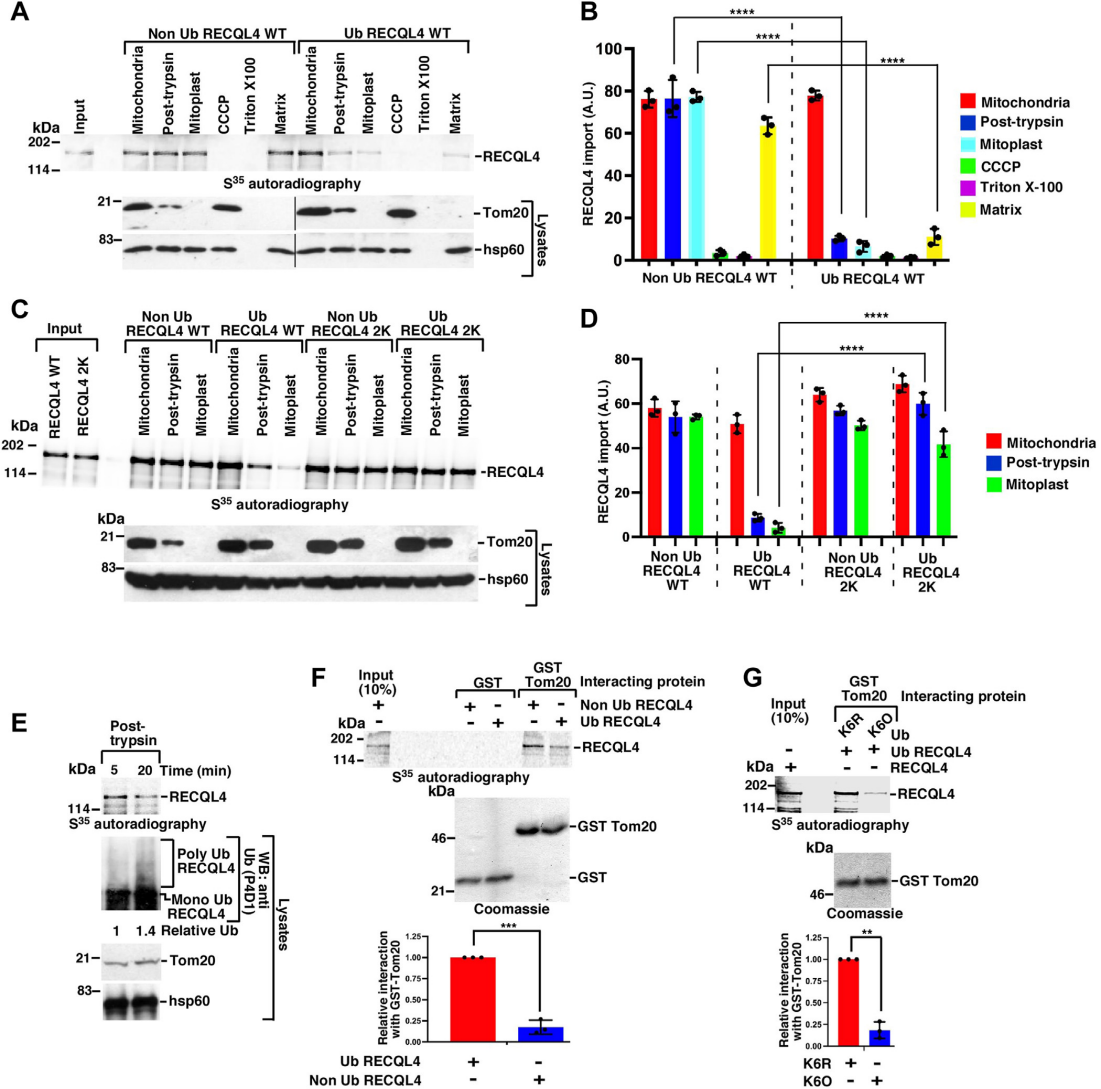
**Ubiquitylation of RECQL4 negatively regulates its mitochondrial entry.***A* and *B*, ubiquitylated Flag-tagged RECQL4 enters mitochondria with increased efficiency. *A*, *in vitro* ubiquitylation of Flag RECQL4 was carried out either with MITOL WT or MITOL CD (as in Fig. 2*A*). The S^35^ methionine-labeled product of the ubiquitylation reactions were used in mitochondrial import assay carried out with the indicated mitochondrial fractions. The extent of entry in each fraction was detected using autoradiography (*top*). The purity of each mitochondrial fraction was analyzed using the indicated antibodies (*bottom*). *B*, quantitation of (*A*), from three replicates. *C* and *D*, RECQL4 2K mutant shows mitochondrial import. *C*, *in vitro* import assay, its subsequent detection, and the quality control of the mitochondrial fractions were carried out same as (*A*). Ubiquitylated and nonubiquitylated Flag-tagged RECQL4 WT or Flag-tagged RECQL4 2K mutant were used. *D*, quantitation of (*C*), from three replicates. *E*, extent of ubiquitylation negatively regulates the entry of RECQL4 in the mitochondria. (*Bottom*) MITOL-dependent *in vitro* ubiquitylation assay of Flag RECQL4 was carried out for either 5 or 20 min. Western blots were done with the indicated antibodies. (*Top*) Differentially ubiquitylated RECQL4 was used in mitochondrial import assay as in (*A*) and the entry of RECQL4 into the post-trypsin mitochondrial fraction was detected by autoradiography. Three replicates were carried out and the same result was obtained. *F*, Tom20 interacts more with nonubiquitylated RECQL4. (*Top*) *In vitro* ubiquitylation reactions of S^35^ methionine-radiolabeled RECQL4 were carried out using either MITOL WT (termed Ub RECQL4) or MITOL CD (termed Non Ub RECQL4). *In vitro* interactions were carried out using Ub RECQL4 or Non Ub RECQL4 with bound GST or GST-Tom20. The bound radioactivity was detected using autoradiography. (*Middle*) Coomassie gel shows the levels of GST or GST Tom20 used in the *in vitro* interaction. (*Bottom*) Quantitation of (*top*), from three replicates. *G*, RECQL4 ubiquitylated by K6 linkage interacts poorly with Tom20. (*Top*) *In vitro* ubiquitylation reactions were carried out as (*F*) except either K6R or K6O ubiquitin was used. *In vitro* interaction was carried out using RECQL4 ubiquitylated by either K6R or K6O and GST Tom20. Bound radioactivity was detected using autoradiography. (*Middle*) Coomassie gel shows the level of GST Tom20 used in the *in vitro* interaction. (*Bottom*) Quantitation of (*top*), from three replicates.

We wanted to investigate whether MITOL-dependent ubiquitylation of RECQL4 affects its mitochondrial import. Hence we carried out *in vitro* import assays with ubiquitylated or nonubiquitylated RECQL4 (Fig. S3*H*). We observed that nonubiquitylated RECQL4 enters the mitochondrial matrix with higher efficiency compared with MITOL-ubiquitylated RECQL4 (Fig. 3, *A* and *B*). It is pertinent to note that nonubiquitylated RECQL4 enters the mitochondrial matrix, where mtDNA replication is known to occur (26). To further understand how ubiquitylation affects RECQL4 mitochondrial import, import assays were carried out with ubiquitylated and nonubiquitylated forms of either RECQL4 WT or RECQL4 2K. RECQL4 2K entered the mitoplast with better efficiency compared with RECQL4 WT (Fig. 3, *C* and *D*).

Furthermore, we wanted to determine the mechanism by which ubiquitylation affects mitochondrial import. For this purpose, MITOL-dependent *in vitro* ubiquitylation reactions of RECQL4 were carried out for 5 min or 20 min (Fig. 3*E*, middle panel) and the ubiquitylation reaction products were used in the import assay. RECQL4 ubiquitylated for 5 min entered the mitochondrial matrix better than RECQL4 ubiquitylated for 20 min (Fig. 3*E*, top panel), indicating a reciprocal correlation exists between the extent of RECQL4 ubiquitylation and mitochondrial import. *In vitro* interactions revealed that nonubiquitylated RECQL4 interacted better with Tom20 compared with ubiquitylated RECQL4 (Fig. 3*F*, the extent of ubiquitylation shown in Fig. S3*H*). Next, we wanted to investigate the role of K6-linked ubiquitylation on RECQL4 vis-à-vis its interaction with Tom20. So *in vitro* ubiquitylation assays of RECQL4 were carried out in the presence of either K6O or K6R variants of ubiquitin (Fig. S3*I*), and these products were used for *in vitro* interaction with GST or GST-Tom20. GST Tom20 interacted better with RECQL4 when it was generated with the K6R variant of ubiquitin (Fig. 3*G*). Together these results indicate that nonubiquitylated RECQL4 entered mitochondria to a better extent.

**Figure 4:**
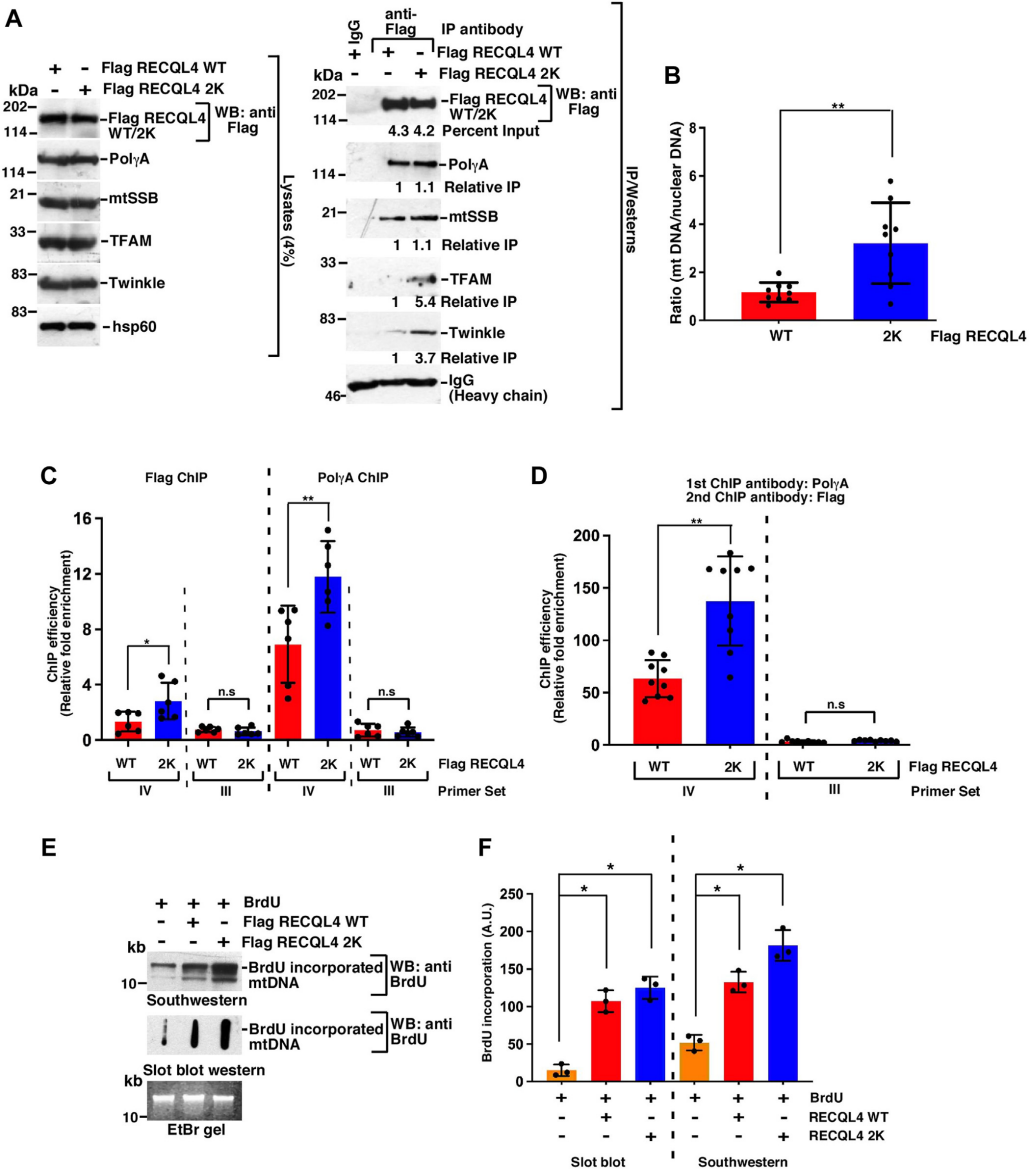
**Nonubiquitylated RECQL4 2K enhances mtDNA replication.***A*, RECQL4 2K interacts better with proteins involved in mitochondrial replication. (*Left*) Whole cell extracts were prepared from HEK293T overexpressing either Flag RECQL4 WT or 2K. Western blot analysis was carried out with the indicated antibodies. (*Right*) Immunoprecipitations were carried out with either anti-Flag antibody or the corresponding IgG control. The immunoprecipitates were probed with the indicated antibodies. Three replicates were carried out and the same result was obtained. *B*, mitochondrial copy number was higher in cells expressing RECQL4 2K. mtDNA copy number was determined in HEK293T cells expressing either Flag RECQL4 WT or 2K. Quantitation is from nine replicates. *C* and *D*, RECQL4 2K enhances the binding of PolγA to the origins of replication in mtDNA D-loop. *C*, mitochondrial chromatin immunoprecipitation (ChIP) or (*D*) Sequential re-ChIP was carried out in HEK293T cells expressing either Flag RECQL4 WT or 2K using either (*C*) anti-Flag or anti-PolγA antibody or (*D*) sequentially anti-PolγA and anti-Flag antibody. The binding of RECQL4 WT/2K and PolγA on mtDNA was determined at two regions of the D-loop using two primer sets. Quantitation is from (*C*) six replicates, (*D*) nine replicates. *E* and *F*, cells expressing RECQL4 2K have enhanced capability of mtDNA replication. *E*, mitochondrial DNA replication in HEK293T cells expressing Flag RECQL4 WT, Flag RECQL4 2K was determined either by (*top*) Southwestern analysis or (*middle*) slot blot Western using anti-BrdU antibody. (*Bottom*) An EtBr gel for NheI digestion of the mtDNA samples shows equal amount of DNA taken for the two assays. *F*, quantitation of the slot blot Western presented in (*E*) from three replicates.

We wanted to decipher whether the enhanced entry of nonubiquitylated RECQL4 and PolγA helped them to carry out their mitochondrial functions with greater efficiency. To test this RECQL4 immunoprecipitations were carried out in cells expressing either RECQL4 WT or 2K. RECQL4 2K interacted better with all the main factors essential for mtDNA replication (namely, PolγA, mtSSB, TFAM, and Twinkle) (Fig. 4*A*). Furthermore, cells expressing RECQL4 2K had higher mitochondrial copy number (Fig. 4*B*), probably as a consequence of better interaction of RECQL4 2K with TFAM (Fig. 4*A*). To determine whether RECQL4 2K had better ability in potentiating PolγA function, mitochondrial chromatin immunoprecipitation assay was performed with an antibody against PolγA in cells expressing either Flag-tagged RECQL4 WT or 2K. Both RECQL4 WT and 2K could potentiate the binding of PolγA to mtDNA in the D-loop region, which contained the mitochondrial origins of replication (primer set IV), and not to an adjacent control region (primer set III). Importantly, the binding of PolγA was significantly more when RECQL4 2K was present. Parallel mitochondrial chromatin immunoprecipitation with anti-Flag antibody also indicated greater binding of RECQL4 2K specifically in the region amplified by primer set IV (Fig. 4*C*). Sequential re-ChIP analysis also indicated that PolγA remained bound to RECQL4 2K with greater efficiency at the mitochondrial origins of replication (Fig. 4*D*). Compared with RECQL4 WT, the presence of RECQL4 2K increased mtDNA replication (determined by the incorporation of BrdU within the mtDNA), as verified by both Southwestern and slot blot Western analysis (Fig. 4, *E* and *F*).

### Ubiquitylation negatively regulates the mitochondrial functions of RTS mutants

**Figure 5:**
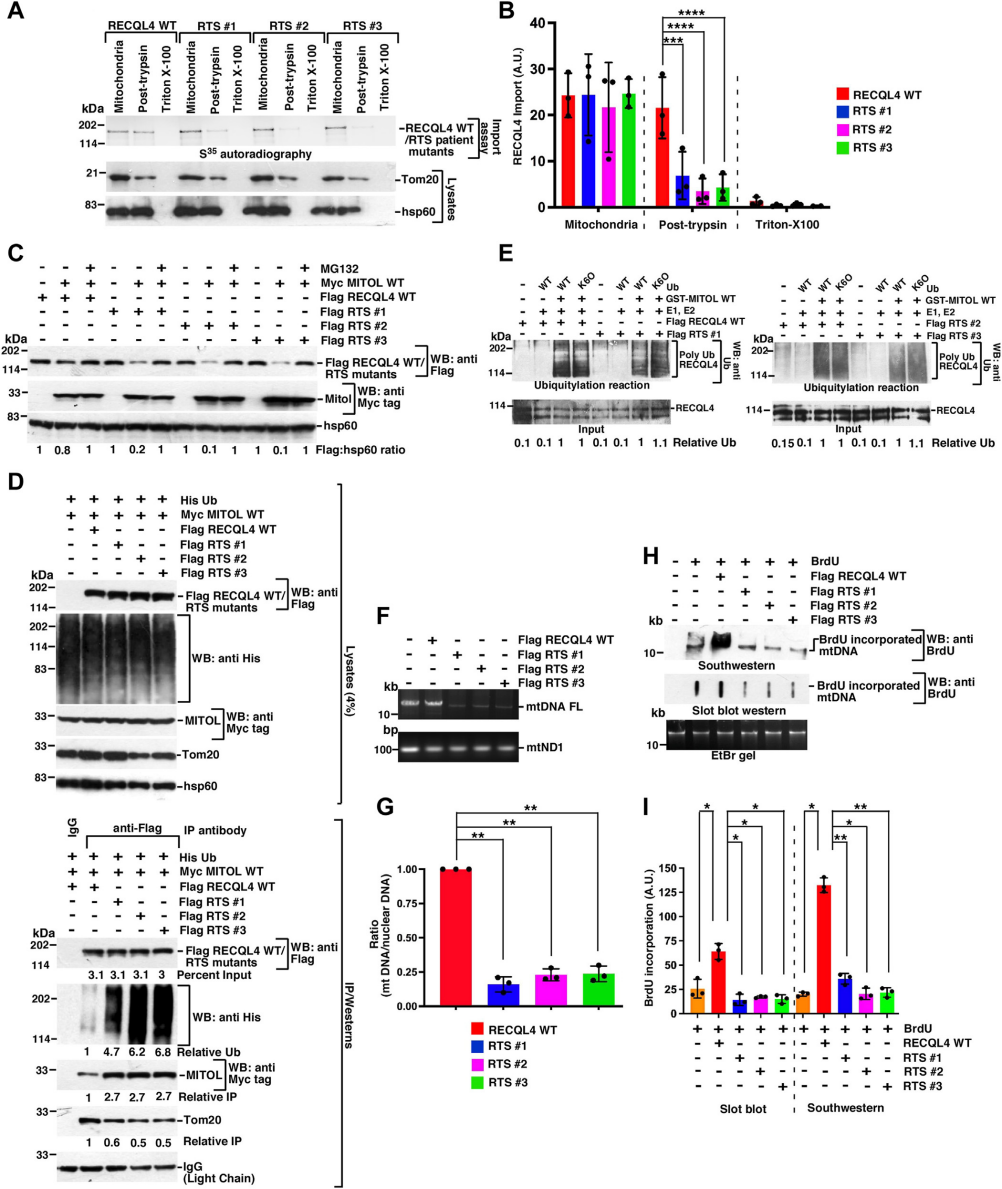
**RTS patient mutants have compromised mitochondrial entry and functions.***A* and *B*, RTS patient mutants have compromised mitochondrial entry. *A*, (*top*) mitochondrial import assay was carried out using the indicated mitochondrial fractions. S^35^ methionine-radiolabeled RECQL4 WT, RTS #1, RTS #2, RTS #3 were used. The extent of entry in each fraction was detected using autoradiography. (*Bottom*) The purity of each mitochondrial fraction was analyzed using Western blot analysis with antibodies against Tom20, hsp60. *B*, quantitation of (*A*), from three replicates. *C*, RTS patient mutants have enhanced rate of proteasomal degradation. Whole cell extracts were made from HEK293T cells overexpressing (for 12 h) Myc MITOL WT, Flag RECQL4 WT, Flag RTS #1, Flag RTS #2, Flag RTS #3. Cells were grown either in absence or presence of MG132 for 5 h. Western blot analysis was carried out with the indicated antibodies. Three replicates were carried out and the same result was obtained. *D*, RTS patient mutants are hyperubiquitylated. (*Top*) Whole cell extracts were made from HEK293T cells transfected with His-tagged ubiquitin (His-Ub), Myc MITOL WT, Flag RECQL4 WT, Flag RTS #1, Flag RTS #2, Flag RTS #3 for 12 h. Western blot analysis was carried out with the indicated antibodies. (*Bottom*) Immunoprecipitations were carried out with anti-Flag antibody (or the corresponding IgG), and the immunoprecipitates were probed with the indicated antibodies. Three replicates were carried out and the same result was obtained. *E*, RTS mutants are ubiquitylated *via* K6 linkage. MITOL-dependent *in vitro* ubiquitylation reactions were carried out using S^35^-radiolabeled Flag RECQL4 WT, Flag RTS #1, Flag RTS #2, Flag RTS #3 as the substrate and Ub WT or Ub K6O as the ubiquitin moiety. Western blots were carried out with anti-ubiquitin antibodies to detect the ubiquitylated products. Input indicates the amount of RECQL4 protein used in each ubiquitylation reaction. Three replicates were carried out and the same result was obtained. *F* and *G*, cells expressing RTS patient mutants have lower mtDNA integrity. *F*, mtDNA integrity was measured using long-range mtDNA amplification assays in HEK293T cells expressing Flag RECQL4 WT, Flag RTS #1, Flag RTS #2, Flag RTS #3. Amplification of mtND1 (100-bp amplicon) was used as a control. *G*, quantitation of (*F*) from three replicates. *H* and *I*, cells expressing RTS mutants have lesser capability of mtDNA replication. *H*, mitochondrial DNA replication in HEK293T cells expressing Flag RECQL4 WT, Flag RTS #1, Flag RTS #2, Flag RTS #3 were determined either by (*top*) Southwestern analysis or (*middle*) slot blot Western using anti-BrdU antibody. (*Bottom*) An EtBr gel for NheI digestion of the mtDNA samples shows equal amount of DNA taken for the two assays. *I*, quantitation of (*H*) from three replicates.

To understand the physiological significance of enhanced import of nonubiquitylated RECQL4 into the mitochondrial matrix, we generated three common missense mutations observed in patients with RTS (Table S1), using the *in vitro*–coupled transcription and translation system (Fig. S4*A*). *In vitro* mitochondrial import assays were carried out with RECQL4 WT and the three mutants revealed that only the wildtype version entered into mitochondria (*i.e.*, post-trypsin fraction) (Fig. 5, *A* and *B*). Next we wanted to determine the *in vivo* ubiquitylation status of RECQL4 WT and the three RTS mutants. The Flag-tagged versions were expressed in HEK293T cells, taking care that their expression was approximately 2.5-fold more compared with endogenous RECQL4 (Fig. S4, *B* and *C*). While expression of MITOL degraded all the RECQL4 variants, the turnover of all three RTS mutants was more compared with RECQL4 WT (Fig. 5*C*). Indeed, the three RTS mutants were hyperubiquitylated compared with RECQL4 WT (Fig. 5*D*). *In vitro* ubiquitylation with WT and K6O ubiquitin variants indicated that, like RECQL4 WT, the three RTS mutants are hyperubiquitylated *via* K6-specific linkage (Fig. 5*E*).

Wildtype RECQL4 is known to localize to mitochondria and maintain mtDNA integrity (7, 8). Therefore, we wanted to determine how mtDNA replication and repair efficiency gets affected in cells expressing wildtype or mutant RECQL4. Using a quantitative PCR-based long-range mtDNA amplification assay that assess both DNA replication and repair we found that the three RTS mutants-expressing cells were highly compromised in this aspect compared with RECQL4 WT (Fig. 5, *F* and *G*). Furthermore, Southwestern and slot blot analysis revealed that BrdU incorporation into mtDNA was significantly enhanced in cells expressing RECQL4 WT (Fig. 5, H and I), thereby indicating cells expressing RTS mutants have decreased mitochondrial repair efficiency and compromised mtDNA polymerization.

**Figure 6:**
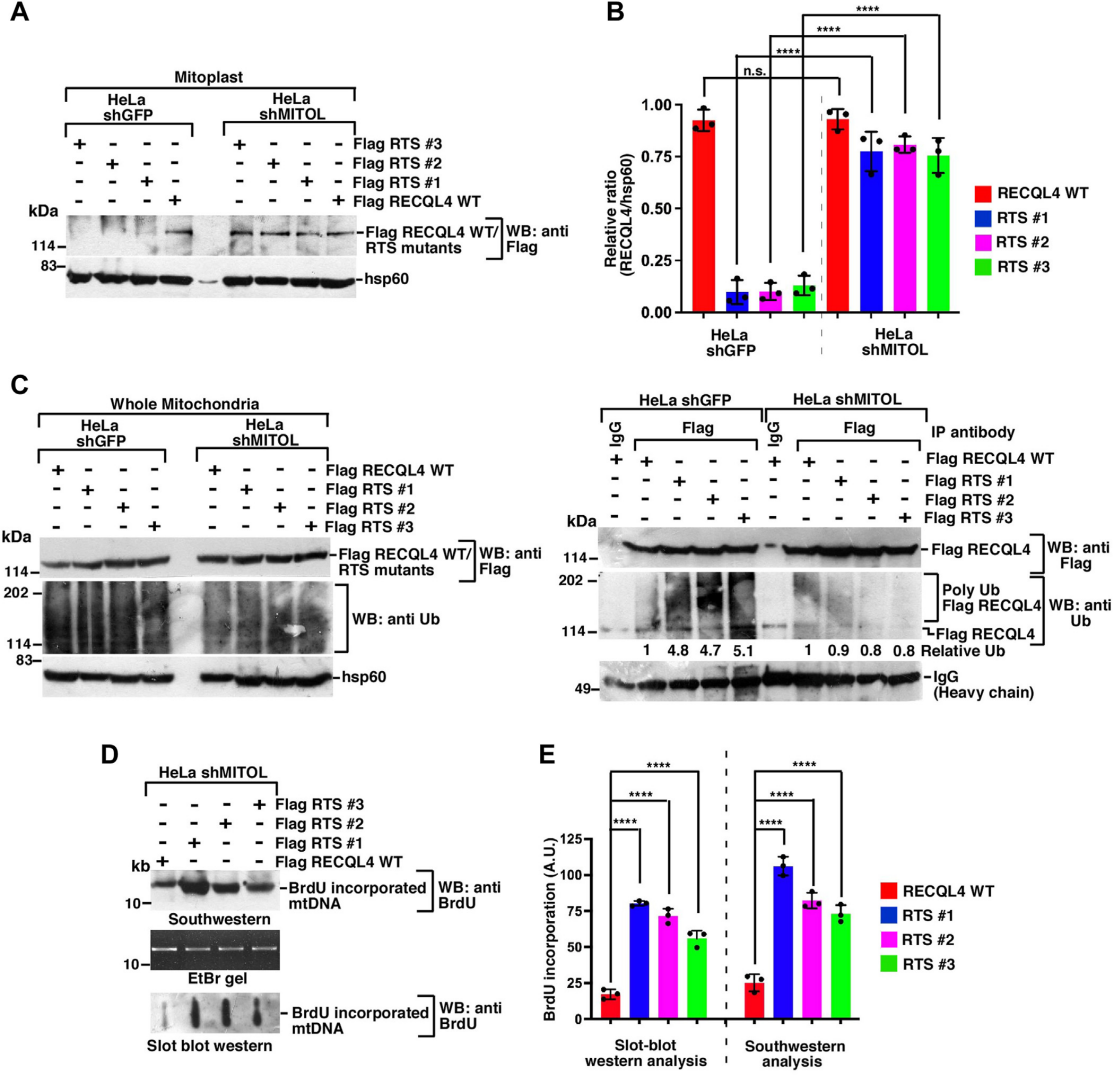
**RTS mutants can be reactivated by depleting MITOL.***A*, HeLa shMITOL cells show increased entry of RTS mutants. Mitoplasts were isolated from HeLa shGFP and HeLa shMITOL cells expressing Flag RECQL4 WT, Flag RTS #1, Flag RTS #2, Flag RTS #3. Western blot analysis was carried out with the indicated antibodies. *B*, the relative levels of RECQL4 to hsp60 have been quantitated from three replicates. *C*, RTS mutants undergo the same level of ubiquitylation as RECQL4 WT in cells lacking MITOL. (*Left*) Whole mitochondrial lysates were isolated from HeLa shGFP and HeLa shMITOL cells expressing Flag RECQL4 WT, Flag RTS #1, Flag RTS #2, Flag RTS #3. Western blot analysis was carried out with the indicated antibodies. (*Right*) Using the above lysates immunoprecipitations were carried out with anti-Flag antibody (or the corresponding IgG). The immunoprecipitates were probed with the indicated antibodies. Three replicates were carried out and the same results were obtained in each case. *D* and *E*, RTS mutants enhance mtDNA replication in cells lacking MITOL. *D*, mitochondrial DNA replication in HeLa shMITOL cells expressing Flag RECQL4 WT, Flag RTS #1, Flag RTS #2, Flag RTS #3 was determined either by (*top*) Southwestern analysis or (*bottom*) slot blot Western using anti-BrdU antibody. (*Middle*) An EtBr gel for NheI digestion of mtDNA shows equal amount of DNA taken for both the assays. *E*, quantification of (*D*) from three replicates.

To validate whether hyperubiquitylation by MITOL indeed affects the mitochondrial import and subsequently mtDNA replication, we used isogenic HeLa cells, which either express or do not express MITOL. We verified that both endogenous RECQL4 (Fig. S5*A*) and overexpressed Flag RECQL4 (Fig. S5*B*) accumulated in the mitochondria. Next, we checked the ability of RECQL4 WT or the three RTS mutants to enter inside the mitochondria. Like RECQL4 WT, the three RTS mutants were present in the mitoplast in HeLa shMITOL cells (Fig. 6, *A* and *B*). To determine whether the enhanced entry into the mitoplast of the RTS mutants was due to alteration in their ubiquitylation status, *in vivo* ubiquitylation assays were carried out in both HeLa shGFP and HeLa shMITOL cells. In contrast to HeLa shGFP cells, the levels of ubiquitylation of the three RTS mutants were drastically reduced in HeLa shMITOL cells. Indeed, upon MITOL ablation RECQL4 WT and RTS mutants showed equivalent level of ubiquitylation (Fig. 6*C*, right). Once inside the mitochondria of HeLa shMITOL cells, all the three RTS mutants were able to potentiate PolγA-dependent mtDNA replication (8), as observed in both Southwestern and slot blot analysis (Fig. 6, *D* and *E*).

### RTS mutants induce mitophagy

**Figure 7:**
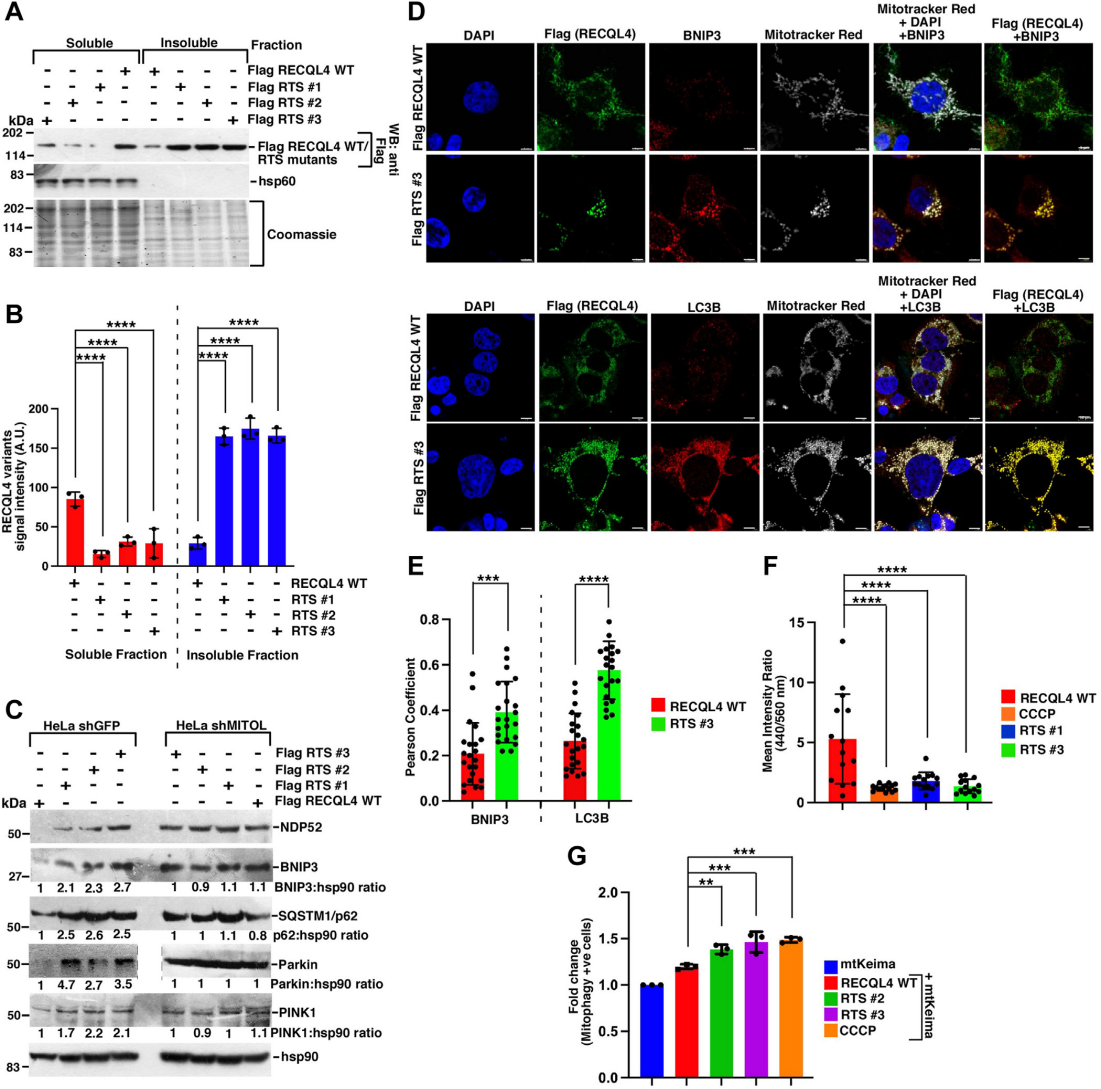
**Cells expressing RTS mutants undergo mitophagy.***A* and *B*, RTS mutants accumulate in the insoluble fraction. *A*, soluble and insoluble fractions were made from HEK293T cells expressing Flag RECQL4 WT, Flag RTS #1, Flag RTS #2, Flag RTS #3 followed by Western analysis with the indicated antibodies. Coomassie gel staining shows the equal amounts of soluble and insoluble fractions used. *B*, quantitation of (*A*) from three replicates. *C*, mitophagy is upregulated in cells expressing RTS mutants. Lysates were made from HeLa shGFP and HeLa shMITOL cells expressing Flag RECQL4 WT, Flag RTS #1, Flag RTS #2, Flag RTS #3. Western blots were carried out with the indicated antibodies. Three replicates were carried out and the same result was obtained. *D* and *E*, BNIP3 and LC3B are upregulated in cells expressing RTS #3. HEK293T cells expressing Flag RECQL4 WT, Flag RTS #3 were treated with MitoTracker Red for 4 h. *D*, immunofluorescence was carried out with antibodies against Flag, BNIP3 (*top*), LC3B (*bottom*). DAPI was used to visualize DNA. The scale bar represents 5 μm (*top*), 10 μm (*bottom*). *E*, Pearson’s coefficient obtained from (*D*) for colocalization of Flag RECQL4 WT, Flag RTS #3 with BNIP3 and LC3B from 22 cells spread over three replicates. *F* and *G*, mt-Keima assay indicates that RTS mutants induce mitophagy. *F*, immunofluorescence was carried out in HEK293T cells expressing mt-Keima alone or with Flag RECQL4 WT or Flag RTS #1 or Flag RTS #3. Cell treated with CCCP was used as positive control. Quantitation of the ratio of mt-Keima signals at 440 nm and 560 nm from 22 cells spread over three replicates. *G*, same as (*F*) except the quantitation was based on a flow cytometry–based mt-Keima assay from five replicates.

Having established that ubiquitylation of RTS mutants affects their import and consequently mtDNA replication, we next wanted to determine the fate of the cells expressing these proteins. MITOL is known to be an important member for cellular quality control and act on proteins that induce toxicity and form aggregates (27, 28, 29). We wanted to determine whether the RTS mutants are enhanced in the detergent-insoluble fraction indicating the propensity to form aggregate-like structures. Equivalent levels of RECQL4 WT and RTS mutants were expressed in HEK293T cells (Fig. S4, *B* and *C*). The three RTS mutants were present to a much greater extent in the insoluble fraction compared with RECQL4 WT (Fig. 7, *A* and *B*).

Hyperfusion of mitochondria is a stress response and has been correlated with cells trying to protect themselves from mitophagy through steric hindrance (30, 31), which if left unrectified leads to degradation of damaged mitochondria or mitophagy (32). Hence we wanted to determine whether expression of RTS mutants potentiated the cells toward mitophagy. Since MITOL itself regulates mitophagy (33), the lack of MITOL induced the expression of multiple mitophagy markers, NDP52, BNIP3, p62, Parkin, and PINK1 (Fig. S6*A*). We further found that expression of RTS mutants (but not RECQL4 WT) increased the expression of the mitophagy markers only in HeLa shGFP cells and not in HeLa shMITOL cells (Fig. 7*C*). Enhanced levels of mitophagy receptor BNIP3 and marker for autophagic activity LC3 were observed in cells that express RTS #3 mutant (Fig. 7*D*). Quantitative analysis revealed increased colocalization of RTS #3 mutant with either BNIP3 or LC3B in comparison with RECQL4 WT, as revealed by Pearson coefficient (Fig. 7*E*). To monitor the difference in the extent of mitophagy between cells expressing RECQL4 WT and the RTS mutants, we used mt-Keima, a fluorescence-based imaging method that involves quantitative measurement (34). The uncoupler carbonyl cyanide m-chlorophenyl hydrazone, known to induce mitophagy in human cells, was used as a positive control. mt-Keima has its excitation maximum at shorter wavelength (around 440 nm) at physiological pH, which shifts to longer wavelength (560 nm) after mitophagy induction. We used two different detection methods involving mt-Keima, by immunofluorescence (Figs. 7*F* and S6*B*) and *via* flow cytometry (Figs. 7*G* and S6*C*). In both the assays mitophagy was significantly enhanced in cells expressing RTS mutants and CCCP-treated cells when compared with cells expressing RECQL4 WT (Figs. 7, *F* and *G* and S6, *B* and *C*). Taken together, the data indicate that the three RTS mutants are present in the insoluble fractions and cells overexpressing these mutants induce mitophagy.

## Discussion

**Figure 8:**
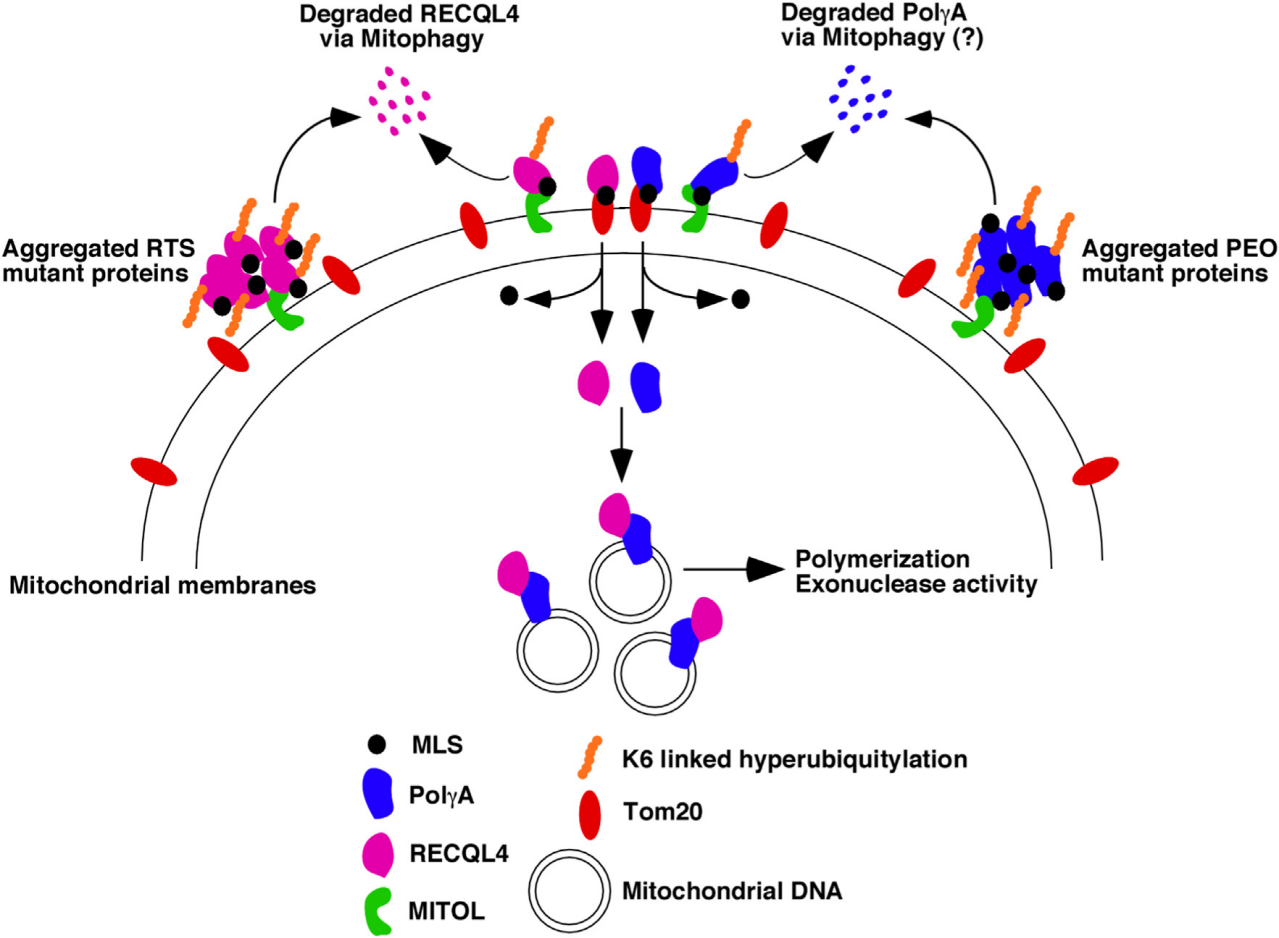
**Mechanism of RECQL4 entry into mitochondria.** RECQL4 has key roles in mitochondrial DNA replication. Mutations in RECQL4 lead to Rothmund–Thomson syndrome (RTS). Wildtype RECQL4 is targeted by MITOL, an E3 ligase present in the outer mitochondrial membrane. MITOL ubiquitylates wildtype RECQL4 at specific residues *via* K6 linkage. Ubiquitylation prevents RECQL4 from entering into mitochondria as the ubiquitylated proteins bind inefficiently with Tom20. Nonubiquitylated RECQL4 enters into the mitochondrial matrix where it binds to mitochondrial polymerase, PolγA. This interaction enhances the binding of PolγA to mtDNA and thereby promotes mitochondrial DNA replication. All the three tested RTS mutants formed aggregate-like structures, which allows them to be recognized and hyperubiquitylated by MITOL, which targets the mutated RECQL4 proteins for degradation and the cells for mitophagy. The lack of entry of these RTS mutants into mitochondria leads to diminished mitochondrial DNA replication and thereby may contribute to the clinical manifestation of RTS. Incidentally PolγA also enters mitochondria by a similar mechanism. Mutations in PolγA contribute significantly to progressive external ophthalmoplegia (PEO). Half of the tested PEO mutants also accumulate on the outer surface of the mitochondria; however, it is not known whether the cells expressing these mutants undergo mitophagy.

It has been thought as a dogma that proteins with MLS will enter into the mitochondria after proteolytic cleavage of the presequence by the mitochondrial processing peptide (35). However, there is lack of precise knowledge about what decides the cleavage of the presequence of the proteins that are supposed to be targeted into the mitochondria. Recent studies by others (21) and us (this study and (20)) provide us clues that the ubiquitin proteasome system present on the outer surface of the mitochondria acts like a “triage,” which decides how much of the mitochondrially targeted proteins should enter into the organelle at any given point of time (Fig. 8). Hence this system fine-tunes the entry of the proteins and thereby possibly acts as a key regulator of mitochondrial protein import and thereby complements the canonical mechanism *via* MLS.

The main player that seems to be playing a central role in this process is an E3 ligase present on the outer membrane of the mitochondria, MITOL. MITOL is already a known player in controlling mitochondrial dynamics and regulates the interconnectivity between the endoplasmic reticulum and mitochondria (15, 18, 22, 36). Here we provide evidence that MITOL interacts with and ubiquitylates a key protein involved in mtDNA replication, RECQL4, which negatively regulates its entry into the mitochondrial matrix and thereby affects its function as an accessory factor to the sole mitochondrial polymerase, PolγA, during mtDNA replication (7, 8). Since the sites on which MITOL ubiquitylates RECQL4 have been validated, we were able to demonstrate that ,when RECQL4 is nonubiquitylated (like when cells express RECQL4 2K mutant, which cannot be ubiquitylated, or when MITOL itself is depleted), the mitochondrial replication is enhanced.

It is known that MITOL ubiquitylates misfolded proteins, eliminates them, and thereby maintains mitochondrial homeostasis (27, 28, 29, 37). We provide evidence that all three tested RTS patient mutants are highly ubiquitylated by MITOL, which prevents them from binding effectively with Tom20 and entering the mitochondrial matrix. We also noted that all three tested RTS mutants form insoluble aggregate-like structures and subsequently cells expressing these mutants undergo mitophagy.

It is interesting to note that, unlike the reported K48 or K63 linkages (27, 29, 38), RECQL4 undergoes ubiquitylation by MITOL *via* K6 linkage. Interestingly the other key protein involved in mitochondrial replication, PolγA, is also ubiquitylated by MITOL *via* the same K6 linkage (20). K6-linked ubiquitination by another mitochondrial E3 ligase PARKIN has been shown to be important for mitophagy (39, 40). Thus, MITOL and PARKIN seem to act in a complementary manner to maintain mitochondrial quality control.

It is perhaps more important to understand how the hyperubiquitylation of RTS mutant proteins, their aggregate formation, and mitophagy affect the pathophysiology of these patients. We had earlier demonstrated that lack of RTS leads to insertions of a significant number of mutations and polymorphisms in the mitochondrial genomes of the RTS patient fibroblasts (8). Using isogenic lines and also RTS patient fibroblasts it was demonstrated that multiple mitochondrial functions were altered in these cells, which ultimately led to diminished oxidative phosphorylation and increased aerobic glycolysis–mediated enhanced invasive capabilities (9). It is interesting to note that developmentally controlled mitophagy promotes a metabolic switch toward glycolysis, which in turn contributes to cellular differentiation (41). It has been reported that mitophagy is an HIF-1-dependent adaptive metabolic response to hypoxia (42). An important factor controlling hypoxia-induced mitophagy is BNIP3/Nix (43). BNIP3 is induced in cells expressing RTS mutants and undergoing mitophagy. Thus ubiquitylation of RECQL4 by MITOL leads to their lack of entry into mitochondria affecting mtDNA replication and integrity. Cells expressing the RECQL4 mutants undergo insoluble aggregate formation leading to mitophagy. All the above aspects affect mitochondrial homeostasis. These factors coupled with RECQL4’s known functions in the nucleus (1, 5, 44) altogether may contribute to the cancer predisposition phenotype seen in patients with RTS. However, more knowledge needs to be generated about the dynamics of MITOL’s interaction with RECQL4 (both for wildtype and RTS mutants) vis-à-vis its ubiquitylation, aggregate-like structure formation, and binding to Tom20. Future work will also shed light about the signal(s) on RECQL4 that allows its interaction and ubiquitylation by MITOL.

## Experimental procedures

### Reagents

The antibodies used in this study have been listed in Table S2. All plasmids used have been reported in Table S3. All the reagents used in this study (including chemicals, recombinant proteins, cell lines, siRNAs, and kits) are mentioned in Table S4. Primers used for different assays are mentioned in Table S5. The mutants used in this study were generated by site-directed mutagenesis.

### Cell culture treatments and immunofluorescence

The cells were transfected with either siRNA or plasmid using Lipofectamine 2000. The transfections were carried out for 6 h according to the manufacturer’s protocol. In each well of the six-well cluster, 0.5 to 1 μg of the plasmid DNA or 200 pmoles of the respective siRNAs was used (unless otherwise specified in the figure legend). The cell lysates were made 24 h post transfection when degradation was supposed to be studied. However, to study only ubiquitylation the lysates were made at an earlier time point (16–18 h post transfection). MG132 (10 μM) treatment was done for the last 5 h of the cell growth. CCCP treatment was for 3 h. Soluble and insoluble fractions were prepared as described (20). For experiment involving benzonase, the lysates were incubated with benzonase (100 unit/ml) on ice for 2 h in a dedicated buffer (50 mM Tris-HCl, pH 8.0 and 1 mM MgCl_2_). For the immunofluorescence experiments, a published protocol was followed (7). In brief, the cells were grown on sterilized 18-μm square cover glasses in 6-well clusters. Cells were fixed in 1X PBS containing 4% formaldehyde for 20 to 30 min. Postfixation cells were permeabilized by treatment with 1X PBS containing 0.1% Triton X-100 for 5 to 10 min and then blocked using 10% chicken serum in 1X PBS containing 0.1% Tween-20 for 30 min. After incubations with primary and secondary antibodies, the cells growing on cover slips were washed three times with 1X PBS containing 0.1% Tween-20 for 5 min each. The coverslips were mounted on the glass slides using a mounting medium with DAPI. Imaging was carried out either in an LSM 510 Meta or in an LSM 980 system with Airyscan 2 (Carl Zeiss). The laser lines used were Argon 458/477/488/514 nm (For FITC), DPSS 561 nm (for Texas Red), He Ne 633 nm (for Alexa Fluor 633), and Blue Diode Laser 405 nm (for DAPI). Pearson coefficient was calculated using ImageJ using the JACoP plugin. Both the colocalization factor and Pearson coefficient were calculated for 100 cells across three biological replicates.

### Mitochondrial fractionation and import assay

Mitochondria were isolated from HEK293T cells exactly as described (20). For import assay RECQL4 WT and the three RTS mutants were *in vitro* transcribed and translated in presence of ^35^S methionine using the T7 Quick Coupled Transcription/Translation System according to the manufacturer’s protocol. The radiolabeled *in vitro*–transcribed and translated products were incubated with the isolated mitochondria and the import assay was carried out as described (20). At the end of the import of the radiolabeled products into different fractions, each sample was divided equally into two parts—one half was used for autoradiography and the other half was used for Western analysis to detect the integrity of the fractions using antibodies to hsp60 and Tom20.

### Protein purification

All proteins were purified from *Escherichia coli* using BL21-CodonPlus-RP competent cells. Protein inductions were always carried out using 1 mM of isopropyl-1-thio-β-D-galactopyranoside (IPTG) and growing the cultures at 200 rpm, 18 °C for 6 h. All other processes were carried out as described (20). Post dialysis, the purity of the samples was checked in a Coomassie-stained gel in the presence of bovine serum albumin standards for relative estimation of their concentrations. The proteins were aliquoted in a storage buffer (25% glycerol, 25 mM Tris pH 7.5, and 140 mM NaCl) and stored in −80 °C freezer until further use.

For purification of proteins from mammalian cells, Flag- or Myc-tagged proteins were overexpressed for 24 to 48 h in HEK293T cells. Depending on the transfection efficiency and the size of the target protein the time post transfection was standardized. Postincubation lysates were prepared using M2 lysis buffer, and expression was checked by Western blot analysis using anti-Flag or anti-Myc tag antibodies. Immunopurification of the tagged proteins was carried out as described (20).

### Immunoblotting and immunoprecipitation

For immunoblotting, the whole cell lysates were prepared using M2 lysis buffer (50 mM Tris pH7.4, 150 mM NaCl, 0.5 mM EDTA, 1% Triton X-100, 10% glycerol, 1X PIC and 1 mM PMSF). For Western blotting, equal amounts of the whole cell lysates were run in 10% SDS-PAGE gels and transferred to the nitrocellulose membrane, which was incubated overnight at 4 °C with the respective primary antibodies. Next day, the membrane was incubated with the respective secondary antibodies for 1 to 2 h and developed using a chemiluminescent detection system. All immunoprecipitations and pull-downs with GST-tagged proteins were carried out as described (20). The extent of immunoprecipitation has been quantitated and is presented as “Relative Immunoprecipitation.” Relative Immunoprecipitation is defined as the extent of immunoprecipitation with respect to the input. To determine quantitatively what percentage of the total protein was immunoprecipitated from the total input, “Percent Input” has been indicated. Percent Input is defined as the percentage of the immunoprecipitated protein with respect to the input for that protein.

### Ubiquitylation and deubiquitylation assays

Protocols for both *in vitro* and in cell ubiquitylation for PolγA by MITOL have recently been described (45). *In vitro* deubiquitylation assays using USP30 were carried out as described in (20, 46). For both the ubiquitylation and deubiquitylation assays, the protocols were adopted for RECQL4 (using either wildtype or CD or 2K variants) as the substrate and MITOL as the E3 ligase. For deubiquitylation assays, USP30 (0.8 μM) was used, either simultaneously with MITOL (called simultaneous reaction) or after completion of MITOL dependent ubiquitylation (called sequential reaction). In the sequential reaction, the deubiquitylation reaction was continued for 90 min more. During *in vitro* ubiquitylation assays, apart from wildtype ubiquitin, KR and KO mutants of ubiquitin have also been used. In KR mutants, the lysine at the particular position was changed to arginine while in KO mutants only the particular lysine was retained while all other lysine residues were changed to arginines. In the NoK mutant all the seven lysines in ubiquitin have been mutated. The extent of ubiquitylation has been quantitated and is presented as “Relative Ubiquitylation.” Relative Ubiquitylation is defined as the extent of ubiquitylation with respect to the input.

### mtDNA copy number

HEK293T cells were transfected with the indicated plasmids. For mtDNA copy number analysis, 48 h post transfection cells were collected and mtDNA was extracted using QiAmp DNA mini kit according to manufacturer’s protocol. Quantitative PCR analysis was carried out to check mtDNA copy number using β-globin as the nuclear gene and cytochrome b as the mitochondrial gene. The nDNA/mtDNA ratio was analyzed as mentioned in (47).

### Measurement of mtDNA replication and repair

HEK293T cells were transfected with either RECQL4 WT or the mutants. Twenty-four hours post transfection cells were treated with BrdU and incubation was continued for 16 h. From these cells mtDNA was isolated using the mtDNA isolation kit, according to the manufacturer’s protocol. With the isolated mtDNA, both Southwestern and slot blot analysis were carried out as described (20). To determine the integrity of mtDNA a long-range mtDNA amplification assay was carried out using 20 ng of the isolated mtDNA template, LongAmp Taq DNA polymerase, and the primers (described in Table S5). For all the above assays, the products were separated by electrophoresis on a 1% agarose gel and autoradiographed or visualized by ethidium bromide staining.

### mt-Keima assay using flow cytometry

HEK293T cells were transfected with either RECQL4 WT or mutants for 48 h. For mt-Keima-based mitophagy detection assay, the cells were run on a FACS Canto II (BD Bioscience) flow cytometer. Voltage was established using the negative control (transfected but unstained cells) and the positive control (cells treated with CCCP). The debris was separated from cells by gating based on forward to side scatter area. Single cells were identified based on forward and side scatter plots. Within the single cell population mt-Keima^+^ cells were identified based on untransfected control. Dual excitations at 488 nm and 561 nm were carried out, while 610-nm emission filter was used for ratiometric mt-Keima measurement. For each condition 100,000 events were collected and the ratio of mitophagy-positive cells to neutral cells was calculated using FlowJo.

### Statistical analysis and quantitation

All data are presented as mean ± SD. The statistical significance is presented as ∗ *p* < 0.05, ∗∗ *p* < 0.01, ∗∗∗ *p* < 0.001, ∗∗∗∗ *p* < 0.001. The statistical analysis employed for every experiment and the exact *p* values have been shown in Table S6. All quantitation of immunoblots were carried out after ImageJ analysis.

## Data availability

All data pertaining to this article (raw data for Western analysis, quantification data, files pertaining to imaging) have been deposited in a public database and the URL is https://data.mendeley.com/v1/datasets/h9r6wn3k83/draft?a=370d3430-ab8a-4fd5-933d-f50fd810b13e.

## Supporting information

This article contains supporting information.

## Conflict of interest

The authors declare that they have no conflicts of interest with the contents of this article.

## References

[1] Lu H., Davis A.J. (2021). Human RecQ helicases in DNA double-strand break repair. Front. Cell Dev. Biol..

[2] Siitonen H.A., Sotkasiira J., Biervliet M., Benmansour A., Capri Y., Cormier-Daire V. (2009). The mutation spectrum in RECQL4 diseases. Eur. J. Hum. Genet..

[3] Hussain M., Krishnamurthy S., Patel J., Kim E., Baptiste B.A., Croteau D.L. (2021). Skin abnormalities in disorders with DNA repair defects, premature aging, and mitochondrial dysfunction. J. Invest. Dermatol..

[4] Larizza L., Roversi G., Volpi L. (2010). Rothmund-Thomson syndrome. Orphanet J. Rare Dis..

[5] Luong T.T., Bernstein K.A. (2021). Role and regulation of the RECQL4 family during genomic integrity maintenance. Genes (Basel).

[6] Croteau D.L., Rossi M.L., Canugovi C., Tian J., Sykora P., Ramamoorthy M. (2012). RECQL4 localizes to mitochondria and preserves mitochondrial DNA integrity. Aging Cell.

[7] De S., Kumari J., Mudgal R., Modi P., Gupta S., Futami K. (2012). RECQL4 is essential for the transport of p53 to mitochondria in normal human cells in the absence of exogenous stress. J. Cell Sci..

[8] Gupta S., De S., Srivastava V., Hussain M., Kumari J., Muniyappa K. (2014). RECQL4 and p53 potentiate the activity of polymerase gamma and maintain the integrity of the human mitochondrial genome. Carcinogenesis.

[9] Kumari J., Hussain M., De S., Chandra S., Modi P., Tikoo S. (2016). Mitochondrial functions of RECQL4 are required for the prevention of aerobic glycolysis-dependent cell invasion. J. Cell Sci..

[10] Dennerlein S., Wang C., Rehling P. (2017). Plasticity of mitochondrial translation. Trends Cell Biol..

[11] den Brave F., Gupta A., Becker T. (2021). Protein quality control at the mitochondrial surface. Front. Cell Dev. Biol..

[12] Alsayyah C., Ozturk O., Cavellini L., Belgareh-Touze N., Cohen M.M. (2020). The regulation of mitochondrial homeostasis by the ubiquitin proteasome system. Biochim. Biophys. Acta Bioenerg..

[13] Schulte U., den Brave F., Haupt A., Gupta A., Song J., Muller C.S. (2023). Mitochondrial complexome reveals quality-control pathways of protein import. Nature.

[14] Shiiba I., Takeda K., Nagashima S., Yanagi S. (2020). Overview of mitochondrial E3 ubiquitin ligase MITOL/MARCH5 from molecular mechanisms to diseases. Int. J. Mol. Sci..

[15] Nakamura N., Kimura Y., Tokuda M., Honda S., Hirose S. (2006). MARCH-V is a novel mitofusin 2- and Drp1-binding protein able to change mitochondrial morphology. EMBO Rep..

[16] Xu S., Cherok E., Das S., Li S., Roelofs B.A., Ge S.X. (2016). Mitochondrial E3 ubiquitin ligase MARCH5 controls mitochondrial fission and cell sensitivity to stress-induced apoptosis through regulation of MiD49 protein. Mol. Biol. Cell.

[17] Yonashiro R., Ishido S., Kyo S., Fukuda T., Goto E., Matsuki Y. (2006). A novel mitochondrial ubiquitin ligase plays a critical role in mitochondrial dynamics. EMBO J..

[18] Park Y.Y., Cho H. (2012). Mitofusin 1 is degraded at G2/M phase through ubiquitylation by MARCH5. Cell Div..

[19] Park Y.Y., Lee S., Karbowski M., Neutzner A., Youle R.J., Cho H. (2010). Loss of MARCH5 mitochondrial E3 ubiquitin ligase induces cellular senescence through dynamin-related protein 1 and mitofusin 1. J. Cell Sci..

[20] Hussain M., Mohammed A., Saifi S., Khan A., Kaur E., Priya S. (2021). MITOL-dependent ubiquitylation negatively regulates the entry of PolgammaA into mitochondria. PLoS Biol..

[21] Phu L., Rose C.M., Tea J.S., Wall C.E., Verschueren E., Cheung T.K. (2020). Dynamic regulation of mitochondrial import by the ubiquitin system. Mol. Cell.

[22] Sugiura A., Nagashima S., Tokuyama T., Amo T., Matsuki Y., Ishido S. (2013). MITOL regulates endoplasmic reticulum-mitochondria contacts via Mitofusin2. Mol. Cell.

[23] Dietschy T., Shevelev I., Pena-Diaz J., Huhn D., Kuenzle S., Mak R. (2009). p300-mediated acetylation of the Rothmund-Thomson-syndrome gene product RECQL4 regulates its subcellular localization. J. Cell Sci..

[24] Kulathu Y., Komander D. (2012). Atypical ubiquitylation - the unexplored world of polyubiquitin beyond Lys48 and Lys63 linkages. Nat. Rev. Mol. Cell Biol..

[25] Michel M.A., Swatek K.N., Hospenthal M.K., Komander D. (2017). Ubiquitin linkage-specific affimers reveal insights into K6-linked ubiquitin signaling. Mol. Cell.

[26] Mazunin I.O., Levitskii S.A., Patrushev M.V., Kamenski P.A. (2015). Mitochondrial matrix processes. Biochemistry (Mosc).

[27] Sugiura A., Yonashiro R., Fukuda T., Matsushita N., Nagashima S., Inatome R. (2011). A mitochondrial ubiquitin ligase MITOL controls cell toxicity of polyglutamine-expanded protein. Mitochondrion.

[28] Yonashiro R., Sugiura A., Miyachi M., Fukuda T., Matsushita N., Inatome R. (2009). Mitochondrial ubiquitin ligase MITOL ubiquitinates mutant SOD1 and attenuates mutant SOD1-induced reactive oxygen species generation. Mol. Biol. Cell.

[29] Yoo Y.S., Park Y.Y., Kim J.H., Cho H., Kim S.H., Lee H.S. (2015). The mitochondrial ubiquitin ligase MARCH5 resolves MAVS aggregates during antiviral signalling. Nat. Commun..

[30] Gomes L.C., Di Benedetto G., Scorrano L. (2011). During autophagy mitochondria elongate, are spared from degradation and sustain cell viability. Nat. Cell Biol..

[31] Rambold A.S., Kostelecky B., Elia N., Lippincott-Schwartz J. (2011). Tubular network formation protects mitochondria from autophagosomal degradation during nutrient starvation. Proc. Natl. Acad. Sci. U. S. A..

[32] Friedman J.R., Nunnari J. (2014). Mitochondrial form and function. Nature.

[33] Shiiba I., Takeda K., Nagashima S., Ito N., Tokuyama T., Yamashita S.I. (2021). MITOL promotes cell survival by degrading Parkin during mitophagy. EMBO Rep..

[34] Sun N., Malide D., Liu J., Rovira, Combs C.A., Finkel T. (2017). A fluorescence-based imaging method to measure *in vitro* and *in vivo* mitophagy using mt-Keima. Nat. Protoc..

[35] Wiedemann N., Pfanner N. (2017). Mitochondrial machineries for protein import and assembly. Annu. Rev. Biochem..

[36] Karbowski M., Neutzner A., Youle R.J. (2007). The mitochondrial E3 ubiquitin ligase MARCH5 is required for Drp1 dependent mitochondrial division. J. Cell Biol..

[37] Kim S.H., Park Y.Y., Yoo Y.S., Cho H. (2016). Self-clearance mechanism of mitochondrial E3 ligase MARCH5 contributes to mitochondria quality control. FEBS J..

[38] Shi H.X., Liu X., Wang Q., Tang P.P., Liu X.Y., Shan Y.F. (2011). Mitochondrial ubiquitin ligase MARCH5 promotes TLR7 signaling by attenuating TANK action. PLoS Pathog..

[39] Durcan T.M., Tang M.Y., Perusse J.R., Dashti E.A., Aguileta M.A., McLelland G.L. (2014). USP8 regulates mitophagy by removing K6-linked ubiquitin conjugates from parkin. EMBO J..

[40] Ordureau A., Sarraf S.A., Duda D.M., Heo J.M., Jedrychowski M.P., Sviderskiy V.O. (2014). Quantitative proteomics reveal a feedforward mechanism for mitochondrial PARKIN translocation and ubiquitin chain synthesis. Mol. Cell.

[41] Esteban-Martinez L., Sierra-Filardi E., McGreal R.S., Salazar-Roa M., Marino G., Seco E. (2017). Programmed mitophagy is essential for the glycolytic switch during cell differentiation. EMBO J..

[42] Zhang H., Bosch-Marce M., Shimoda L.A., Tan Y.S., Baek J.H., Wesley J.B. (2008). Mitochondrial autophagy is an HIF-1-dependent adaptive metabolic response to hypoxia. J. Biol. Chem..

[43] Bellot G., Garcia-Medina R., Gounon P., Chiche J., Roux D., Pouyssegur J. (2009). Hypoxia-induced autophagy is mediated through hypoxia-inducible factor induction of BNIP3 and BNIP3L via their BH3 domains. Mol. Cell Biol..

[44] Croteau D.L., Popuri V., Opresko P.L., Bohr V.A. (2014). Human RecQ helicases in DNA repair, recombination, and replication. Annu. Rev. Biochem..

[45] Hussain M., Saifi S., Mohammed A., Sengupta S. (2022). Protocol to detect *in vitro* and in cell ubiquitylation of mitochondrial DNA polymerase gamma by mitochondrial E3 ligase MITOL. STAR Protoc..

[46] Rusilowicz-Jones E.V., Jardine J., Kallinos A., Pinto-Fernandez A., Guenther F., Giurrandino M. (2020). USP30 sets a trigger threshold for PINK1-PARKIN amplification of mitochondrial ubiquitylation. Life Sci. Alliance.

[47] Quiros P.M., Goyal A., Jha P., Auwerx J. (2017). Analysis of mtDNA/nDNA ratio in mice. Curr. Protoc. Mouse Biol..

