# Supplementary material for: Hyperubiquitylation of DNA helicase RECQL4 by E3 ligase MITOL prevents mitochondrial entry and potentiates mitophagy
Source: J Biol Chem. 2023 Jul 24;299(9):105087. doi: 10.1016/j.jbc.2023.105087 (PMC10470078; doi:10.1016/j.jbc.2023.105087)
Supplement: Supporting Figures S1–S6 and Tables S1–S6 [file mmc1.pdf]

Supplementary Information

Hyper-ubiquitylation of DNA helicase RECQL4 by E3 ligase MITOL prevents mitochondrial entry and potentiates mitophagy

Mansoor Hussain [2,4], Aftab Mohammed [2,4], Shabnam Saifi [2], Swati Priya [2], Sagar Sengupta [1,2,3]

[1] National Institute of Biomedical Genomics, PO: NSS, Kalyani, 741251, India.
[2] National Institute of Immunology, Aruna Asaf Ali Marg, New Delhi 110067, India.

[4]Contributed equally

[3] Corresponding author:
Sagar Sengupta,
National Institute of Biomedical Genomics,
PO: NSS, Kalyani, 741251,
India.
Email: ssg2@nibmg.ac.in

Running Title: Hyper-ubiquitylation of RECQL4 causes mitophagy

Keywords: Rothmund Thomson Syndrome, mitochondrial replication, RecQ helicases, E3 ligases, autophagy

Supplementary Figures and legends

Figure S1
Hussain et al.

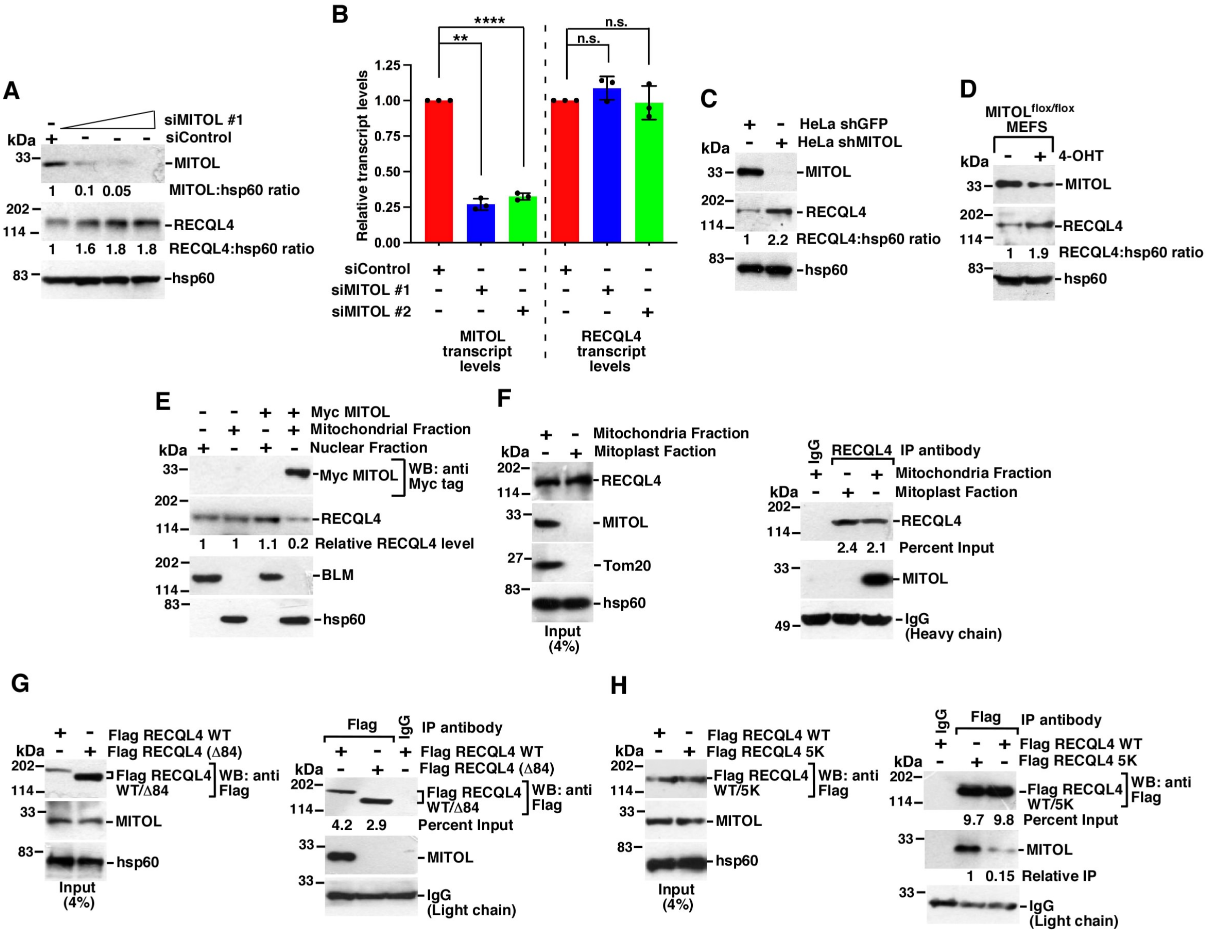

Figure S1: Mitochondrial RECQL4 is a substrate of MITOL

A, B. Ablation of MITOL increases RECQL4 protein and transcript levels. HEK293T cells were transfected with (A) siControl or a gradient of siMITOL #1 (100 pmole, 200 pmole, 300 pmole per well of a 6-well cluster) (B) siControl or siMITOL #1 or siMITOL#2. (A) Whole cell lysates were made and western blotting carried out with the indicated antibodies. (B) RNA was isolated and RT-qPCR carried out to detect the levels of MITOL and RECQL4. The transcript levels of GAPDH was used as control. Data is from three replicates.

C, D. Depletion of MITOL enhanced RECQL4 levels in different experimental systems. Whole cell extracts were made from (C) HeLa shGFP and Hela shMITOL cells or from (D) MITOL$^{flox/flox}$ cells either untreated or treated with 4-OHT for 4 days. Western blot analysis

was carried out with the indicated antibodies. Three replicates were carried out and same result was obtained.

E. Overexpression of MITOL depletes mitochondrial RECQL4. Nuclear and mitochondrial fractions were isolated from HEK293T cells expressing Myc MITOL. Western blot analysis was carried out with the indicated antibodies. Three replicates were carried out and same result was obtained.

F. Interaction of MITOL with RECQL4 occur on the surface of the mitochondria. (Left) Total mitochondria and mitoplast fractions obtained from HEK293T cells were probed with the indicated antibodies. (Right) Immunoprecipations were carried out with anti-RECQL4 antibody (or the corresponding IgG). Immunoprecipitates was probed with the indicated antibodies. Three replicates were carried out and same result was obtained.

G. MLS of RECQL4 is essential for its interaction with MITOL. (Left) Lysates were made from HEK293T cells expressing Flag RECQL4 WT, Flag RECQL4 (Δ84). Western blot analysis was carried out with the indicated antibodies. (Right) Immunoprecipations were carried out with anti-Flag antibody (or the corresponding IgG). Immunoprecipitates was probed with the indicated antibodies. Three replicates were carried out and same result was obtained.

H. Nuclear export of RECQL4 allows it to interact with Tom20. (Left) Lysates were made from HEK293T cells expressing either RECQL4 WT or Flag RECQL4 5K. Western blot analysis was carried out with the indicated antibodies. (Right) Immunoprecipations were carried out with anti-Flag antibody (or the corresponding IgG). Immunoprecipitates were probed with the indicated antibodies. Four replicates were carried out and same result was obtained.

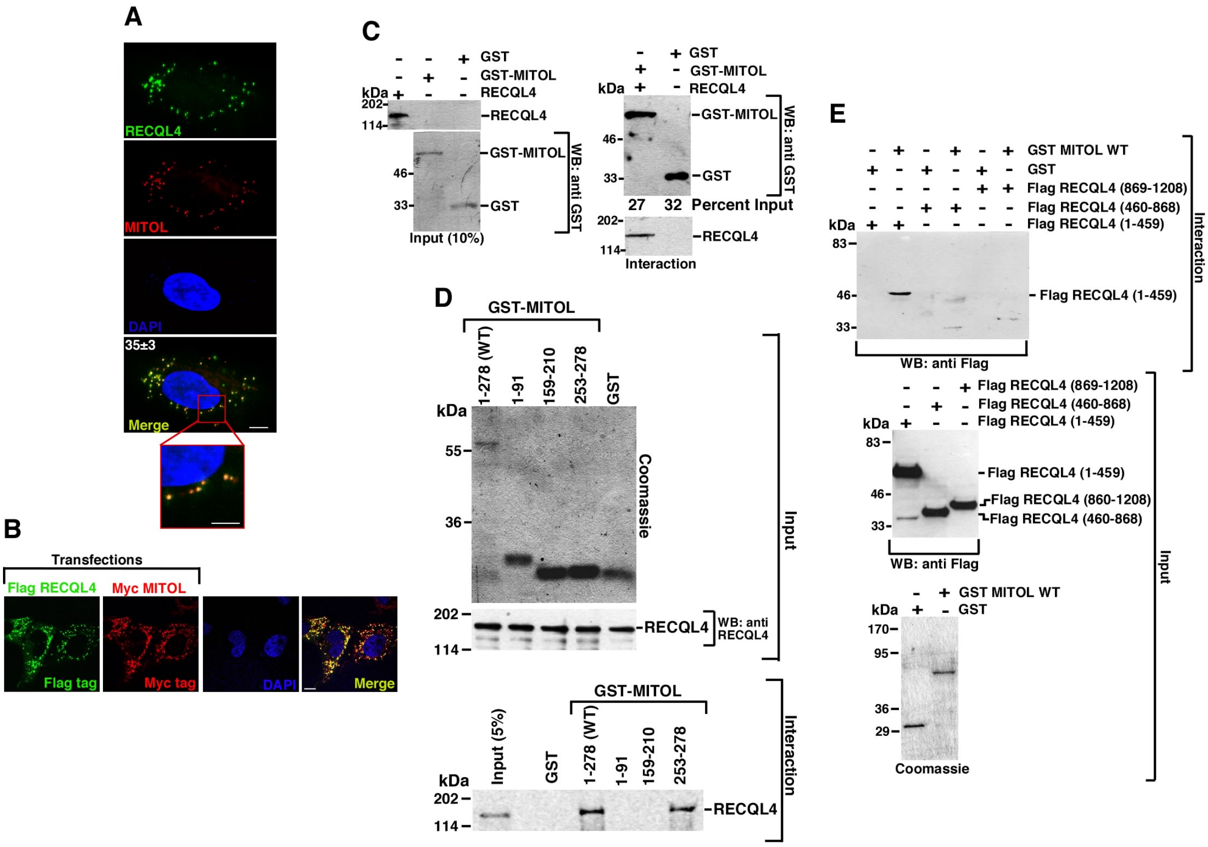

Figure S2: N-terminal of RECQL4 interact with the C-terminal loop region of MITOL

A. Endogenous RECQL4 and MITOL colocalize. Asynchronously growing U-2 OS cells were stained with anti-RECQL4 and anti-MITOL antibodies. Scale bar 5µm. Representative images are shown from two replicates.

B. Exogenously expressed Flag RECQL4 and Myc MITOL colocalize. U-2 OS cells were co-transfected with Flag RECQL4 WT and Myc MITOL. Cells were stained with anti-Flag and anti-Myc tag antibodies. Scale bar 5µm. Representative images are shown from two replicates.

C. RECQL4 interact with GST-MITOL. (Left) Input showing *in vitro* transcribed and translated RECQL4 and bound GST, GST MITOL. (Right) *In vitro* interaction was carried out between bound GST/GST MITOL and RECQL4. The interaction was detected with the indicated antibodies.

D. C-terminal loop of MITOL interacts with RECQL4. (Input, Top) Bound GST or GST tagged MITOL (1-278), MITOL (1-191), MITOL (159-210), MITOL (253-278)] were visualized by Coomassie staining. S^{35} methionine radiolabeled RECQL4 was *in vitro* transcribed and translated and the product was probed with anti-RECQL4 antibody. (Interaction, bottom) Interaction were carried out between S^{35} methionine radiolabeled RECQL4 and bound GST or GST tagged MITOL (1-278), MITOL (1-191), MITOL (159-210), MITOL (253-278). The amount of radiolabeled RECQL4 bound to the GST-tagged proteins was detected by autoradiography. Three replicates were carried out and same result was obtained.

E. N-terminal region of RECQL4 interacts with MITOL. (Interaction, Top) Interactions between bound GST or GST-MITOL and immunopurified Flag tagged RECQL4 (1-459), RECQL4 (460-868), RECQL4 (869-1208) were carried out. The bound protein(s) to the Flag beads were detected with anti-Flag tag antibody. Three replicates were carried out and same result was obtained. (Input, Bottom) Flag tagged RECQL4 proteins [RECQL4 (1-459), RECQL4 (460-868), RECQL4 (869-1208)] were expressed in HEK293T, immunopurified, eluted and detected by anti-Flag antibody. Coomassie gel showing the purity of bound GST, GST MITOL WT.

Figure S3
Hussain et al.

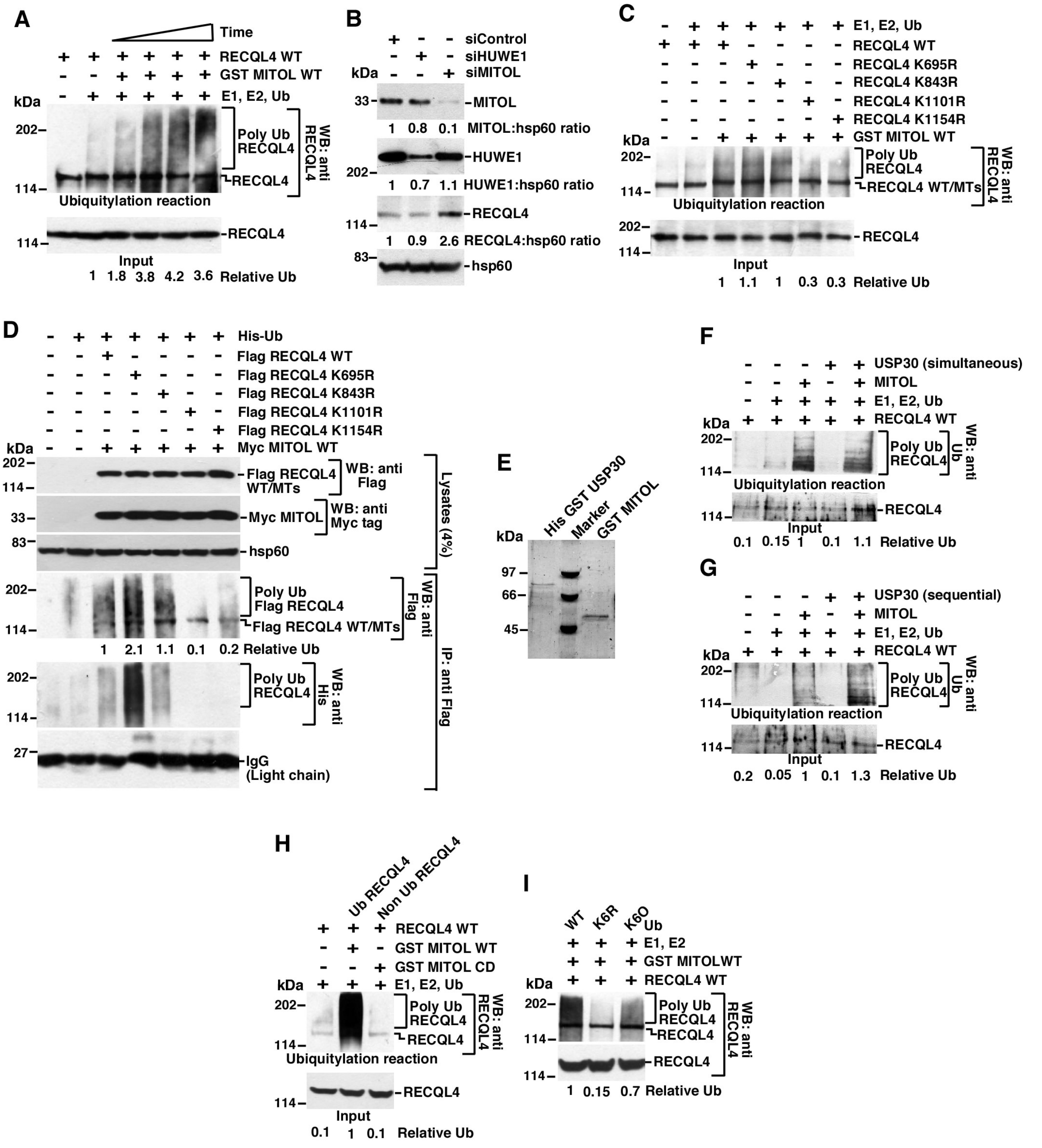

Figure S3: Ubiquitylation of RECQL4 by MITOL occur at specific residues via K6 linkage.

A. Time course of RECQL4 ubiquitylation by MITOL. *In vitro* ubiquitylation reactions were carried out using RECQL4 as the substrate and MITOL WT as the E3 ligase. (Top) The ubiquitylation reactions were carried out for 5min, 10min, 20min, 30min. (Bottom) Input indicates the amount of RECQL4 protein used in each ubiquitylation reaction. In both cases

anti-RECQL4 antibody was used for detection. Three replicates were carried out and same result was obtained.

B. RECQL4 is not a substrate of HUWE1. Lysates were made from HEK293T cells transfected with either siControl or siHUWEI or siMITOL. Western blot analysis was carried out with the indicated antibodies. Three replicates were done and same results were obtained.

C. RECQL4 is ubiquitylated by MITOL at K1101 and K1154 *in vitro*. *In vitro* ubiquitylation reactions were carried out using S^{35} methionine radiolabeled RECQL4 WT and four RECQL4 mutants namely K695R, K843R, K1101R, K1154R as the substrate. Post-reaction, the products were detected by Western blot analysis with anti-RECQL4 antibody. Input indicates the amount of RECQL4 protein used in each ubiquitylation reaction. Three replicates were carried out and same result was obtained.

D. RECQL4 is ubiquitylated by MITOL at K1101 and K1154 *in vivo*. (Top) Whole cell extracts were prepared form HEK293T transfected with His-Ub, Myc MITOL and Flag RECQL4 WT or four RECQL4 mutants namely K695R, K843R, K1101R, K1154R. Western blot analysis was carried out with the indicated antibodies. (Bottom) Immunoprecipitations were carried out with anti-Flag antibody and the immunoprecipitates were probed with antibodies against Flag and His tag. Three replicates were carried out and same result was obtained.

E. Coomassie gel of purified recombinant proteins. Three independent protein preparations for GST, GST MITOL and GST USP30 were used for the experiments. One representative Coomassie shown.

F, G. RECQL4 is not a substrate of USP30 *in vitro*. *In vitro* ubiquitylation reactions were carried out using RECQL4 as the substrate and MITOL WT as the E3 ligase. Recombinant USP30 was added either (F) during the *in vitro* ubiquitylation assay (called simultaneous reaction) or (G) after MITOL mediated *in vitro* ubiquitylation assay (called sequential reaction). Post-reaction, the products were detected by Western blot analysis with the indicated antibodies. Input indicates the amount of RECQL4 used in every reaction. Three replicates were carried out and same result was obtained.

H. Generation of Ub RECQL4 and Non Ub RECQL4**.** *In vitro* ubiquitylation reactions were carried out using S^{35} methionine radiolabeled RECQL4 as the substrate and MITOL WT or CD as the E3 ligase. Post-ubiquitylation, the products were detected by carrying out Western blot analysis with anti-RECQL4 antibodies. The reaction products where RECQL4 were ubiquitylated by MITOL WT were designated as Ub RECQL4. The products obtained when MITOL CD was used were designated as Non Ub RECQL4. Input indicates the amount of RECQL4 used in each condition was determined by western blotting with antibodies against RECQL4. Three replicates were carried out and same result was obtained.

I. RECQL4 was ubiquitylated by Ub K6O and not by Ub K6R. Same as (H) except MITOL dependent ubiquitylation reactions for RECQL4 were carried out with two ubiquitin variants - Ub K6R or Ub K6O. Three replicates were carried out and same result was obtained.

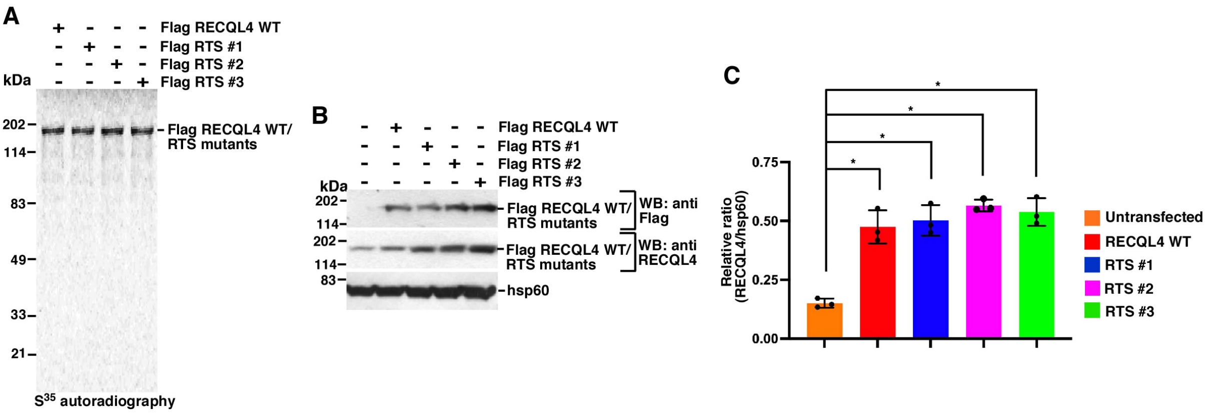

Figure S4: Levels of RECQL4 WT and RTS mutants

A. Levels of RECQL4 WT and RTS mutants. *In vitro* transcribed and translated RECQL4 WT, RTS #1, RTS #2, RTS #3 were subjected to SDS-PAGE and the products were detected by autoradiography. Three replicates were carried out and same result was obtained.

B, C. Relative levels of endogenous and exogenous RECQL4 WT and PEO mutants. (B) Lysates were made from HEK293T cells transfected with Flag RECQL4 WT, Flag RTS #1, Flag RTS #2, Flag RTS #3. Western blot analysis was carried out with the indicated antibodies. (C) The relative levels of RECQL4 to hsp60 have been quantitated from three replicates.

Figure S5
Hussain et al.

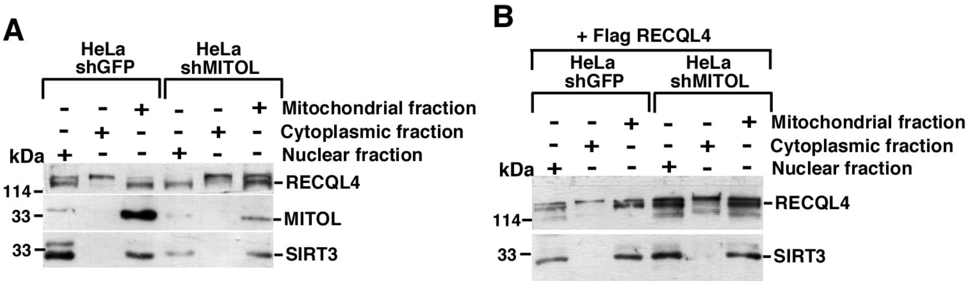

Figure S5: Both endogenous and exogenous RECQL4 localizes to the mitochondria

Nuclear extract, cytoplasmic fraction and mitochondrial fraction were isolated from HeLa shGFP and HeLa shMITOL. The fractionations were carried out from the two cell types which were either (A) asynchronously grown or (B) transfected with Flag RECQL4. For both conditions western analysis were carried out with the indicated antibodies.

Figure S6
Hussain et al.

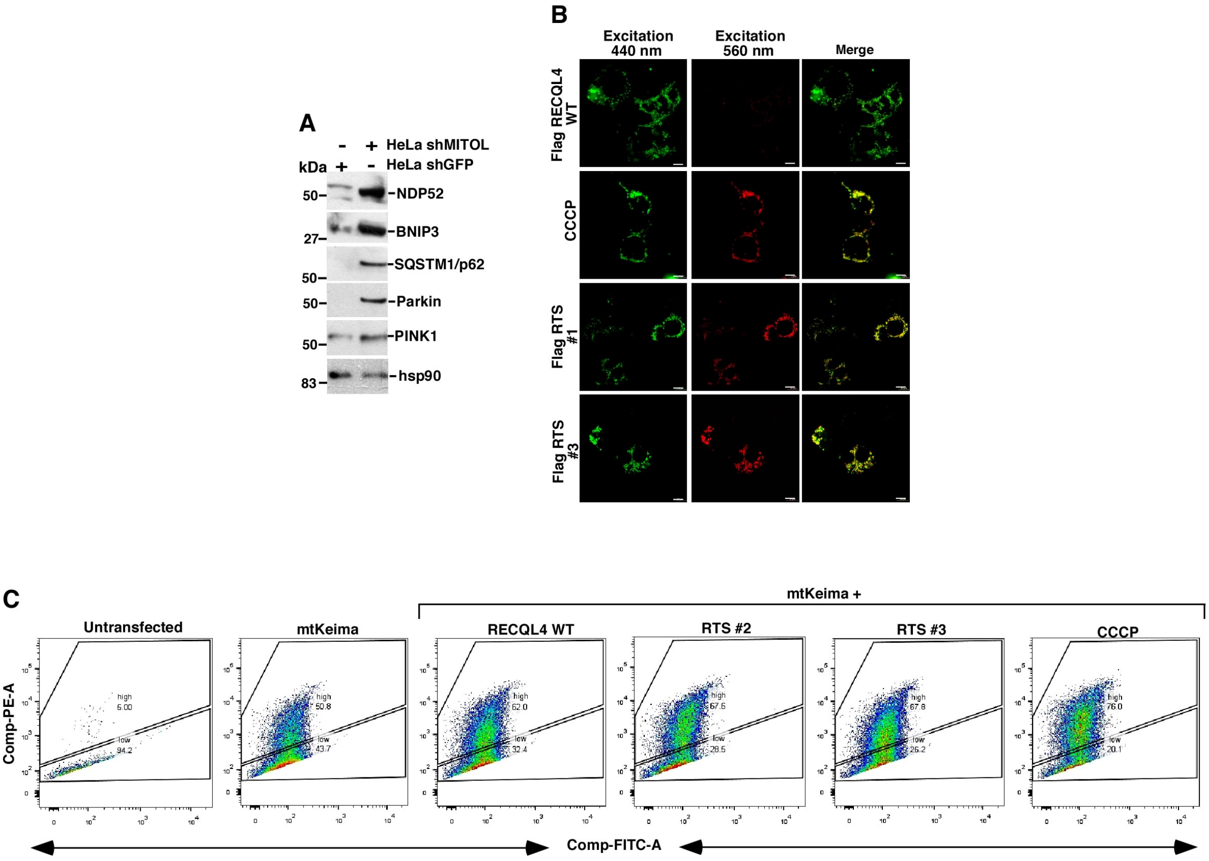

Figure S6: RTS mutants induce mitophagy.

A. Lack of MITOL induce mitophagy. Lysates were made from asynchronously growing HeLa shGFP and HeLa shMITOL. Western analysis were carried out with the indicated antibodies.

B, C. Expression of RTS mutants induce mitophagy. (B) Immunofluorescence was carried out on HEK293T cells expressing either RECQL4 WT, RTS #1, RTS #3 along with mt-Keima. Cells were also treated with CCCP (used as a positive control). Cells were fixed and analysed using confocal microscopy. Scale bar 10μm. (C) Same as (B) except post-experiment HEK293T cells were analysed by flow cytometry. Representative images from three replicates for (B) and five replicates for (C).

Table S1: Details of patient mutations used in the study

RTS patient mutants		
**Identifier**	**Mutation**	**Approximate Molecular Weight**
RTS patient mutant #1 (RTS #1)	P466L	133kDa
RTS patient mutant #2 (RTS #2)	F637S	133kDa
RTS patient mutant #3 (RTS #3)	F697L	133kDa

Table S2: List of antibodies used in the study

Name of antibody	Source	Identifier
Anti-RECQL4 (used for WB, IP)	Santa Cruz Biotechnology	Cat# sc-366840
Anti-RECQL4 (used for WB, IP)	Novus Biologicals	Cat# 25470002; RRID:AB_936259
Anti-RECQL4 (used for IF)	Santa Cruz Biotechnology	Cat# sc-16924, RRID:AB_2253673
Anti-RECQL4 (used for IF)	Yasuhiro Furuichi, GeneCare Research Institute, 200 Kajiwara, Kamakura, 247, Japan	(Kawabe et al., 2000)
Anti-PolγA (used for WB, mt-ChIP)	Santa Cruz Biotechnology	Cat# sc-390634
Anti-MITOL (used for WB)	Shigeru Yanagi (Tokyo University of Pharmacy and Life Sciences, Japan)	(Yonashiro et al., 2006)
Anti-MITOL (used for WB)	Novus Biologicals	Cat# NBP1-59585
Anti-hsp60 (used for WB)	Abcam	Cat# ab87085; RRID:AB_10672924
Anti-hsp60 (used for WB)	Abcam	Cat# ab46798; RRID:AB_881444
Anti-Myc tag (used for WB, IF)	Cell Signaling Technology	Cat#2278; RRID:AB_10693332
Anti-Flag (used for WB, IF)	Sigma-Aldrich	Cat# F1804; RRID:AB_262044
Anti-Flag M2 affinity gel (used for IP)	Sigma-Aldrich	Cat# F2220
Anti-TFAM (used for WB)	Abcam	Cat# ab131607; RRID:AB_11154693
Anti-Twinkle (used for WB)	Abcam	Cat# ab83329; RRID:AB_1859960
Anti-Ub (P4D1) (used for WB)	Santa Cruz Biotechnology	Cat# sc-8017; RRID:AB_628423
Anti-His (used for WB)	Santa Cruz Biotechnology	Cat# sc-8036; RRID:AB_627727
Anti-Tom20 (used for WB)	Santa Cruz Biotechnology	Cat# sc-17764; RRID:AB_628381
Anti-BrdU (used for SBW, SW)	Abcam	Cat# ab1893; RRID: AB_302659
Anti-HUWE1 (used for WB)	Bethyl Laboratories	Cat# A300-486A; RRID: AB_ AB_2264590
Anti-diUbiquitin K6 affimer (used for WB)	Avacta	Cat# AVA00100
Anti-Lon (used for WB)	Novus Biologicals	Cat# H00009361-D01P RRID: AB_2137153
Anti-DRP1	Santa Cruz Biotechnology	Cat# sc-32898

(used for WB)		RRID: AB_2093533
Anti-mtSSB<br>(used for WB)	Sigma-Aldrich	Cat# HPA002866;<br>RRID:AB_1080092
Anti-NDP52<br>(used for WB)	Cell Signaling Technology	Cat# 60732<br>RRID: AB_2732810
Anti-BNIP3<br>(used for WB, IF)	Cell Signaling Technology	Cat# 44060<br>RRID: AB_2799259
Anti-SQSTM1/p62<br>(used for WB)	Cell Signaling Technology	Cat# 8025<br>RRID: AB_10859911
Anti-Parkin<br>(used for WB)	Cell Signaling Technology	Cat# 4211<br>RRID: AB_2159920
Anti-PINK1<br>(used for WB)	Cell Signaling Technology	Cat# 6946<br>RRID: AB_11179069
Anti-LC3B<br>(used for WB, IF)	Cell Signaling Technology	Cat# 3868<br>RRID: AB_2137707
Anti-hsp90<br>(used for WB)	Santa Cruz Biotechnology	Cat# sc-7947<br>RRID: AB_2121235
Anti-mtSSB<br>(used for WB)	Santa Cruz Biotechnology	Cat# sc-34725<br>RRID: AB_2195318
Anti-BLM<br>(used for WB)	Bethyl Laboratories	Cat# A300-110A<br>RRID: AB_2064794

WB: Western blotting
IP: Immunoprecipitation
IF: Immunofluorescence
SBW: Slot blot western
SW: Southwestern

References:

Kawabe, T., Tsuyama, N., Kitao, S., Nishikawa, K., Shimamoto, A., Shiratori, M., Matsumoto, T., Anno, K., Sato, T., Mitsui, Y. et al. (2000). Differential regulation of human RecQ family helicases in cell transformation and cell cycle. *Oncogene* **19**, 4764-72.

Yonashiro, R., Ishido, S., Kyo, S., Fukuda, T., Goto, E., Matsuki, Y., Ohmura-Hoshino, M., Sada, K., Hotta, H., Yamamura, H. et al. (2006). A novel mitochondrial ubiquitin ligase plays a critical role in mitochondrial dynamics. *EMBO J* **25**, 3618-26.

Table S3: List of recombinant DNAs used in the study

Name of the recombinant DNA	Source	Identifier
pA Puro MITOL WT-myc or Myc MITOL WT	Shigeru Yanagi (Tokyo University of Pharmacy and Life Sciences, Japan)	(Yonashiro et al., 2006)
pA Puro MITOL CD-myc or Myc MITOL CD	This study	N/A
pGEX4T-1 MITOL WT or GST MITOL WT	This study	N/A
pGEX4T-1 MITOL CD or GST MITOL CD	This study	N/A
pGEX4T-1 MITOL N ter or GST MITOL (1-91)	Shigeru Yanagi (Tokyo University of Pharmacy and Life Sciences, Japan)	(Sugiura et al., 2013)
pGEX4T-1 MITOL 2nd loop or GST MITOL (159-210)	Shigeru Yanagi (Tokyo University of Pharmacy and Life Sciences, Japan)	(Sugiura et al., 2013)
pGEX4T-1 MITOL C ter or GST MITOL (253-278)	Shigeru Yanagi (Tokyo University of Pharmacy and Life Sciences, Japan)	(Sugiura et al., 2013)
pcDNA4-TO myc-his-B PARKIN WT	Quan Chen (State Key Laboratory of Membrane Biology, China)	(Chen et al., 2017)
pcDNA4-TO myc-his-B MULAN WT	Quan Chen (State Key Laboratory of Membrane Biology, China)	(Chen et al., 2017)
pFLAG-CMV4 RNF185 WT	Quan Chen (State Key Laboratory of Membrane Biology, China)	(Chen et al., 2017)
pFLAG-CMV4 KEAP1 WT	Quan Chen (State Key Laboratory of Membrane Biology, China)	(Chen et al., 2017)
His-Ub	Akhil Banerjea, (National Institute of Immunology, India)	N/A
pGEX-6P-1-ratTom20 (59–126)	Daisuke Kohda (Kyushu University, Japan)	(Saitoh et al., 2007)
pcDNA3-Flag-RECQL4 WT	Present in the lab of corresponding author	(De et al., 2012)

pcDNA3-Flag-RECQL4 WT (Δ84)	Present in the lab of corresponding author	(De et al., 2012)
pcDNA3-Flag-RECQL4 (K695R)	This study	N/A
pcDNA3-Flag-RECQL4 (K843R)	This study	N/A
pcDNA3-Flag-RECQL4 (K1101R)	This study	N/A
pcDNA3-Flag-RECQL4 (K1151R)	This study	N/A
pcDNA3-Flag-RECQL4 (K1101R, K1151R) or Flag-RECQL4 2K	This study	N/A
pcDNA3-Flag-RECQL4 (K376R, K380R, K382R, K385R, K386R) or Flag-RECQL4 5K	Re-generated in the lab of corresponding author	(Dietschy et al., 2009)
pcDNA3-Flag-RECQL4 P466L or Flag RTS #1	This study	N/A
pcDNA3-Flag-RECQL4 F637S or Flag RTS #2	This study	N/A
pcDNA3-Flag-RECQL4 F697L or Flag RTS #3	This study	N/A
pcDNA 3.1 hygro (+) RECQL4 WT	This study	N/A
pcDNA3-Flag-RECQL4 (1-459)	Present in the lab of corresponding author	(De et al., 2012)
pcDNA3- Flag-RECQL4 (460-868)	Present in the lab of corresponding author	(Gupta et al., 2014)
pcDNA3- Flag-RECQL4 (869-1208)	Present in the lab of corresponding author	(Gupta et al., 2014)
His-GST-USP30	David Komander (Addgene plasmid #110744)	(Gersch et al., 2017)
pHAGE-mt-mKeima	Richard Youle (Addgene plasmid #131626)	(Vargas et al., 2019)

Chen, Z., Liu, L., Cheng, Q., Li, Y., Wu, H., Zhang, W., Wang, Y., Sehgal, S. A., Siraj, S., Wang, X. et al. (2017). Mitochondrial E3 ligase MARCH5 regulates FUNDC1 to fine-tune hypoxic mitophagy. *EMBO Rep* **18**, 495-509.

De, S., Kumari, J., Mudgal, R., Modi, P., Gupta, S., Futami, K., Goto, H., Lindor, N. M., Furuichi, Y., Mohanty, D. et al. (2012). RECQL4 is essential for the transport of p53 to mitochondria in normal human cells in the absence of exogenous stress. *J Cell Sci* **125**, 2509-22.

Dietschy, T., Shevelev, I., Pena-Diaz, J., Huhn, D., Kuenzle, S., Mak, R., Miah, M. F., Hess, D., Fey, M., Hottiger, M. O. et al. (2009). p300-mediated acetylation of the Rothmund-Thomson-syndrome gene product RECQL4 regulates its subcellular localization. *J Cell Sci* **122**, 1258-67.

Gersch, M., Gladkova, C., Schubert, A. F., Michel, M. A., Maslen, S. and Komander, D. (2017). Mechanism and regulation of the Lys6-selective deubiquitinase USP30. *Nat Struct Mol Biol* **24**, 920-930.

Gupta, S., De, S., Srivastava, V., Hussain, M., Kumari, J., Muniyappa, K. and Sengupta, S. (2014). RECQL4 and p53 potentiate the activity of polymerase

gamma and maintain the integrity of the human mitochondrial genome. *Carcinogenesis* **35**, 34-45.

Saitoh, T., Igura, M., Obita, T., Ose, T., Kojima, R., Maenaka, K., Endo, T. and Kohda, D. (2007). Tom20 recognizes mitochondrial presequences through dynamic equilibrium among multiple bound states. *EMBO J* **26**, 4777-87.

Sugiura, A., Nagashima, S., Tokuyama, T., Amo, T., Matsuki, Y., Ishido, S., Kudo, Y., McBride, H. M., Fukuda, T., Matsushita, N. et al. (2013). MITOL regulates endoplasmic reticulum-mitochondria contacts via Mitofusin2. *Mol Cell* **51**, 20-34.

Vargas, J. N. S., Wang, C., Bunker, E., Hao, L., Maric, D., Schiavo, G., Randow, F. and Youle, R. J. (2019). Spatiotemporal Control of ULK1 Activation by NDP52 and TBK1 during Selective Autophagy. *Mol Cell* **74**, 347-362 e6.

Yonashiro, R., Ishido, S., Kyo, S., Fukuda, T., Goto, E., Matsuki, Y., Ohmura-Hoshino, M., Sada, K., Hotta, H., Yamamura, H. et al. (2006). A novel mitochondrial ubiquitin ligase plays a critical role in mitochondrial dynamics. *EMBO J* **25**, 3618-26.

Table S4: List of reagents used in the study

Name	Source	Identifier
**Chemicals**		
Cycloheximide	Sigma-Aldrich	Cat# C7698; CAS Number 66-81-9
MG132	Merck	Cat# 474787; CAS Number 133407-82-6
IPTG	Sigma-Aldrich	Cat# I6758; CAS Number 367-93-1
PMSF	Sigma-Aldrich	Cat# P7626; CAS Number 329-98-6
DTT	Sigma-Aldrich	Cat# D0632; CAS Number 3483-12-3
BrdU	Sigma-Aldrich	Cat# B5002; CAS Number 59-14-3
Triton-X-100	Sigma-Aldrich	Cat# T9284; CAS Number 9002-93-1
CCCP	Sigma-Aldrich	Cat# C2759; CAS Number 555-60-2
Benzonase nuclease	Sigma-Aldrich	Cat# E1014-5KU; CAS Number 9025-65-4
**Recombinant proteins**		
Ubiquitin	Enzo	Cat# BML-UW8795-0005; CAS Number 79586
Ubiquitin activating enzyme E1 Ube 1 (human)	Enzo	Cat# BML-UW9410-0050
UbcH5a (human)	Enzo	Cat# BML-UW9050-0100
Ubiquitin (K63O)	Boston Biochem	Cat# UM-HK630
Ubiquitin (K48O)	Boston Biochem	Cat# UM-HK480
Ubiquitin (K6R)	Boston Biochem	Cat# UM-K6R
Ubiquitin (K11R)	Boston Biochem	Cat# UM-K11R
Ubiquitin (K27R)	Boston Biochem	Cat# UM-K27R
Ubiquitin (K29R)	Boston Biochem	Cat# UM-K29R
Ubiquitin (K33R)	Boston Biochem	Cat# UM-K33R
Ubiquitin (K48R)	Boston Biochem	Cat# UM-K48R
Ubiquitin (K63R)	Boston Biochem	Cat# UM-K63R
Ubiquitin No K	Boston Biochem	Cat# UB-NOK
GST MITOL Wildtype (WT)	Present in the lab of corresponding author	(Hussain et al., 2021)
GST MITOL Catalytic Dead (CD)	Present in the lab of corresponding author	(Hussain et al., 2021)
GST Tom20	Present in the lab of	(Hussain et al., 2021)

	corresponding author	
GST MITOL (1-91)	Present in the lab of corresponding author	(Hussain et al., 2021)
GST MITOL (159-210)	Present in the lab of corresponding author	(Hussain et al., 2021)
GST MITOL (253-278)	Present in the lab of corresponding author	(Hussain et al., 2021)
His GST USP30	Addgene plasmid #110744	(Gersch et al., 2017)
**Cell Lines**		
HEK293T	Present in the lab of corresponding author	ATCC Cat# CRL-3216
HeLa shGFP	Shigeru Yanagi (Tokyo University of Pharmacy and Life Sciences, Japan)	(Sugiura et al., 2013)
HeLa shMITOL	Shigeru Yanagi (Tokyo University of Pharmacy and Life Sciences, Japan)	(Sugiura et al., 2013)
GM07532-hTERT (Normal Human Fibroblasts, NHF)	Present in the lab of corresponding author	(Sengupta et al., 2003)
U-2 OS	Present in the lab of corresponding author	ATCC Cat# HTB-96
**Oligonucleotides**		
siRNA sequences for MITOL	Dharmacon	(Yonashiro et al., 2006)
siRNA sequences for HUWE1	Dharmacon	(Peter et al., 2014)
ON-TARGETplus Non-targeting siRNA #1	Dharmacon	Cat # D-001810-01-05

RT-qPCR primers for RECQL4, see Table S5	Sigma-Aldrich	This study
RT-qPCR primers for MITOL, see Table S5	Sigma-Aldrich	(Hussain et al., 2021)
RT-qPCR primers for GAPDH, see Table S5	Sigma-Aldrich	(Hussain et al., 2021)
Primers for Long range mtDNA amplification assay, see Table S5	Sigma-Aldrich	N/A
**Other**		
Fetal bovine Serum	Thermo Fisher Scientific	Cat# 10082147
Advanced DMEM	Thermo Fisher Scientific	Cat# 12491-023
QuikChange II XL Site-Directed Mutagenesis Kit	Agilent	Cat# 200522
Mitochondrial DNA isolation kit	BioVision, Inc.	Cat# K280-50
T7 Quick coupled Transcription/ Translation system	Promega	Cat# L2080
[^{35}S] Methionine	Perkin Elmer	Cat# NEG009T
Trizol reagent	Thermo Fisher Scientific	Cat# 15596026
Reverse Transcriptase Core Kit	Eurogentec	Cat# RT-RTCK-05
Qubit dsDNA HS assay kit	Thermo Fisher Scientific	Cat# Q32851
Lipofectamine 2000	Thermo Fisher Scientific	Cat# 11668019
Complete Protease Cocktail inhibitor	Roche	Cat# 11697498001
BL21-CodonPlus-RP	Agilent	Cat# 230250
Poly-Prep Chromatography column	Biorad	Cat# 73101550
Flag peptide	Sigma-Aldrich	Cat# 3290
LongAmp Taq DNA polymerase	New Englands Biolab	Cat# M0323L
QIAamp DNA Mini Kit	Qiagen	Cat# 51304

Immobilon Western Chemiluminescent HRP substrate	Merck	Cat# WBKLS0500

Reference:

Gersch, M., Gladkova, C., Schubert, A. F., Michel, M. A., Maslen, S. and Komander, D. (2017). Mechanism and regulation of the Lys6-selective deubiquitinase USP30. *Nat Struct Mol Biol* **24**, 920-930.

Hussain, M., Mohammed, A., Saifi, S., Khan, A., Kaur, E., Priya, S., Agarwal, H. and Sengupta, S. (2021). MITOL-dependent ubiquitylation negatively regulates the entry of PolgammaA into mitochondria. *PLoS Biol* **19**, e3001139.

Peter, S., Bultinck, J., Myant, K., Jaenicke, L. A., Walz, S., Muller, J., Gmachl, M., Treu, M., Boehmelt, G., Ade, C. P. et al. (2014). Tumor cell-specific inhibition of MYC function using small molecule inhibitors of the HUWE1 ubiquitin ligase. *EMBO Mol Med* **6**, 1525-41.

Sengupta, S., Linke, S. P., Pedeux, R., Yang, Q., Farnsworth, J., Garfield, S. H., Valerie, K., Shay, J. W., Ellis, N. A., Wasylyk, B. et al. (2003). BLM helicase-dependent transport of p53 to sites of stalled DNA replication forks modulates homologous recombination. *EMBO J* **22**, 1210-1222.

Sugiura, A., Nagashima, S., Tokuyama, T., Amo, T., Matsuki, Y., Ishido, S., Kudo, Y., McBride, H. M., Fukuda, T., Matsushita, N. et al. (2013). MITOL regulates endoplasmic reticulum-mitochondria contacts via Mitofusin2. *Mol Cell* **51**, 20-34.

Yonashiro, R., Ishido, S., Kyo, S., Fukuda, T., Goto, E., Matsuki, Y., Ohmura-Hoshino, M., Sada, K., Hotta, H., Yamamura, H. et al. (2006). A novel mitochondrial ubiquitin ligase plays a critical role in mitochondrial dynamics. *EMBO J* **25**, 3618-26.

Table S5: List of primers used in the study

RT-qPCR primers			
**Identifier**	**Forward primer (5' to 3')**	**Reverse primer (5' to 3')**	**Reference**
RECQL4	TTG AGG AAG ACC CTC CAG	CAT GTT GAG CCG TAC GTA AT	This study and (Hussain et al., 2021)
MITOL	CAG ATC ATG TCT CTG CTA CTC	GGT GTG CCT GTC GTA AAT	
GAPDH	GTC TCC TCT GAC TTC AAC AGC G	ACC ACC CTG TTG CTG TAG CCA A	
**Long range mtDNA amplification assay**			
mtND1	CAC CCA AGA ACA GGG TTT GT	TGG CCA TGG GTA TGT TGT TAA	(Chi et al., 2012)
mtDNA	TGA GGC CAA ATA TCA TTC TGA GGG GC	TTT CAT CAT GCG GAG ATG TTG GAT GG	

Reference:

Chi, Z., Nie, L., Peng, Z., Yang, Q., Yang, K., Tao, J., Mi, Y., Fang, X., Balajee, A. S. and Zhao, Y. (2012). RecQL4 cytoplasmic localization: implications in mitochondrial DNA oxidative damage repair. *Int J Biochem Cell Biol* **44**, 1942-51.

Hussain, M., Mohammed, A., Saifi, S., Khan, A., Kaur, E., Priya, S., Agarwal, H. and Sengupta, S. (2021). MITOL-dependent ubiquitylation negatively regulates the entry of PolgammaA into mitochondria. *PLoS Biol* **19**, e3001139.

Table S6: Statistical analysis performed in this study

Figure number	Statistical Analysis performed	Exact p values
Figure 1A	One-way ANOVA	Bar 1 vs Bar 2, p = 0.0011
Figure 1C	One-way ANOVA	Bar 1 vs Bar 2, p = 0.0023<br>Bar 2 vs Bar 3. p = 0.0111
Figure 1D	One-way ANOVA	Bar 1 vs Bar 2, p = 0.0014<br>Bar 1 vs Bar 3, p = 0.0014
Figure 1E	One-way ANOVA	Bar 1 vs Bar 2, p = 0.0006<br>Bar 1 vs Bar 3, p = 0.0049
Figure 1G	Two-way ANOVA	Row 2, p = 0.0038<br>Row 3, p = 0.0002<br>Row 4, p < 0.0001<br>Row 5, p < 0.0001
Figure 3B	Two-way ANOVA	Bar 2 vs Bar 8, p < 0.0001<br>Bar 3 vs Bar 9, p < 0.0001<br>Bar 6 vs Bar 12, p < 0.0001
Figure 3D	Two-way ANOVA	Bar 5 vs Bar 11, p < 0.0001<br>Bar 6 vs Bar 12, p < 0.0001
Figure 3F	Paired t test (two-tailed)	Bar 1 vs Bar 2, p = 0.0034
Figure 3G	Paired t test (two-tailed)	Bar 1 vs Bar 2, p = 0.0045
Figure 4B	Unpaired t test (two-tailed)	Bar 1 vs Bar 2, p = 0.0027
Figure 4C	One-way ANOVA	Bar 1 vs Bar 2, p = 0.0492<br>Bar 5 vs Bar 6, p = 0.0075
Figure 4D	One-way ANOVA	Bar 1 vs Bar 2, p = 0.0012
Figure 4F	One-way ANOVA	Bar 1 vs Bar 2, p = 0.0313<br>Bar 1 vs Bar 3, p = 0.0466<br>Bar 4 vs Bar 5, p = 0.0294<br>Bar 4 vs Bar 6, p = 0.0256
Figure 5B	Two-way ANOVA	Bar 5 vs Bar 6, p = 0.0004<br>Bar 5 vs Bar 7, p < 0.0001<br>Bar 5 vs Bar 8, p < 0.0001
Figure 5G	One-way ANOVA	Bar 1 vs Bar 2, p = 0.0031<br>Bar 1 vs Bar 3, p = 0.0016<br>Bar 1 vs Bar 4, p = 0.0044
Figure 5I	One-way ANOVA	Bar 1 vs Bar 2, p = 0.0484<br>Bar 2 vs Bar 3, p = 0.0463<br>Bar 2 vs Bar 4, p = 0.0393<br>Bar 2 vs Bar 5, p = 0.0228<br>Bar 6 vs Bar 7, p = 0.0127<br>Bar 7 vs Bar 8, p = 0.0087<br>Bar 7 vs Bar 9, p = 0.0183<br>Bar 7 vs Bar 10, p = 0.0073
Figure 6B	One-way ANOVA	Bar 2 vs Bar 6, p < 0.0001<br>Bar 3 vs Bar 7, p < 0.0001<br>Bar 4 vs Bar 8, p < 0.0001
Figure 6E	Two-way ANOVA	Bar 1 vs Bar 2, p < 0.0001<br>Bar 1 vs Bar 3, p < 0.0001

		Bar 1 vs Bar 4, $p < 0.0001$<br>Bar 5 vs Bar 6, $p < 0.0001$<br>Bar 5 vs Bar 7, $p < 0.0001$<br>Bar 5 vs Bar 8, $p < 0.0001$
Figure 7B	Two-way ANOVA	Bar 1 vs Bar 2, $p < 0.0001$<br>Bar 1 vs Bar 3, $p < 0.0001$<br>Bar 1 vs Bar 4, $p < 0.0001$<br>Bar 5 vs Bar 6, $p < 0.0001$<br>Bar 5 vs Bar 7, $p < 0.0001$<br>Bar 5 vs Bar 8, $p < 0.0001$
Figure 7E	One-way ANOVA	Bar 1 vs Bar 2, $p = 0.0005$<br>Bar 3 vs Bar 4, $p < 0.0001$
Figure 7F	One-way ANOVA	Bar 1 vs Bar 2, $p < 0.0001$<br>Bar 1 vs Bar 3, $p < 0.0001$<br>Bar 1 vs Bar 4, $p < 0.0001$
Figure 7G	One-way ANOVA	Bar 2 vs Bar 3, $p = 0.0084$<br>Bar 2 vs Bar 4, $p = 0.0007$<br>Bar 2 vs Bar 5, $p = 0.0004$
Figure S1B	One-way ANOVA	Bar 1 vs Bar 2, $p = 0.0026$<br>Bar 1 vs Bar 3, $p < 0.0001$
Figure S4C	One-way ANOVA	Bar 1 vs Bar 2, $p = 0.0493$<br>Bar 1 vs Bar 3, $p = 0.0476$<br>Bar 1 vs Bar 4, $p = 0.0102$<br>Bar 1 vs Bar 5, $p = 0.0249$
